# β-Formyl- and β-Vinylporphyrins: Magic Building Blocks for Novel Porphyrin Derivatives †

**DOI:** 10.3390/molecules22081269

**Published:** 2017-07-29

**Authors:** Ana F. R. Cerqueira, Nuno M. M. Moura, Vanda Vaz Serra, M. Amparo F. Faustino, Augusto C. Tomé, José A. S. Cavaleiro, M. Graça P. M. S. Neves

**Affiliations:** 1 Department of Chemistry and QOPNA, University of Aveiro, 3810-193 Aveiro, Portugal; anacerqueira@ua.pt (A.F.R.C.); nmoura@ua.pt (N.M.M.M.); vanda.serra@tecnico.ulisboa.pt (V.V.S.); faustino@ua.pt (M.A.F.F.); actome@ua.pt (A.C.T.); jcavaleiro@ua.pt (J.A.S.C.); 2 Centro de Química Estrutural, Instituto Superior Técnico, Universidade de Lisboa, Av. Rovisco Pais 1, 1049-001 Lisboa, Portugal

**Keywords:** porphyrins, formyl group, vinyl group, methylene active compounds, imines, pericylic reactions

## Abstract

Porphyrins bearing formyl or vinyl groups have been explored as starting materials to prepare new compounds with adequate features for different applications. In this review it is discussed mainly synthetic strategies based on the reaction of *meso*-tetraarylporphyrins bearing those groups at β-pyrrolic positions. The use of some of the obtained porphyrin derivatives for further transformations, namely via pericyclic reactions, is also highlighted.

## 1. Introduction

**Figure 1:**
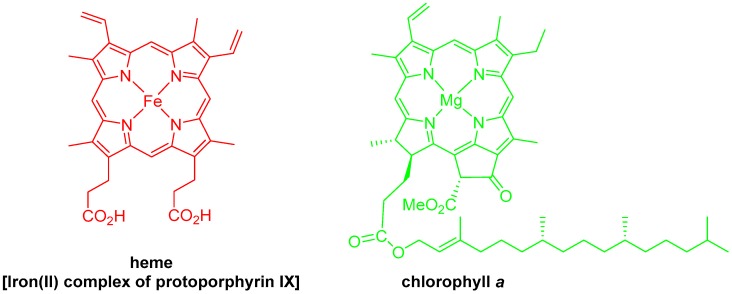
The structures of heme and chlorophyll *a*.

The role of tetrapyrrolic macrocycles like porphyrins and analogues (Figure 1) in vital biological functions such as respiration and photosynthesis, and their unique properties responsible for important applications in different fields, explains why the scientific community maintains high interest in this type of macrocycle.

The contributions of Hans Fischer to the final structure elucidation of protoporphyrin-IX and hemin reported in 1929 and of Woodward in the synthesis of chlorophyll *a* in 1960 are important highlights in the extraordinary evolution observed afterwards with these molecules [1,2,3]. Today, natural and synthetic porphyrin derivatives are recognized as excellent systems to be explored in the development of novel catalysts [4,5,6,7,8], electronic devices [9,10,11,12], sensors [13,14,15,16], dyes for photovoltaic solar cells [17,18,19,20,21,22] and as therapeutic agents [23,24,25,26,27]. In medicine, porphyrin derivatives are being used as photosensitisers in the treatment of oncologic and non-oncologic diseases by photodynamic therapy (PDT) [28,29,30,31,32,33]. More recently, their role as photosensitisers has been extended to inactivate pathogenic microorganisms in blood components [34], food [35], surfaces [36] and wastes [37,38,39,40,41,42,43].

**Figure 2:**
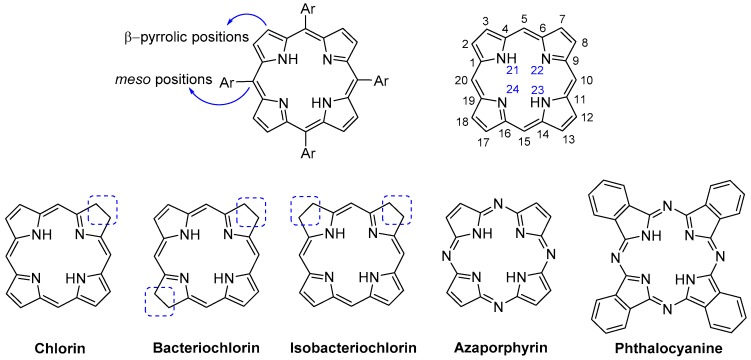
General structure of *meso*-tetraarylporphyrins and the IUPAC numbering of the porphyrinic nucleus. The structures of other nucleus that will be mentioned throughout the manuscript are also depicted.

All these applications are strongly dependent on the availability of compounds with adequate and specific structural features. This explains the high number of studies related with the preparation and modification of natural and synthetic porphyrin derivatives or analogues (Figure 2). In particular, synthetic porphyrins like *meso*-tetraarylporphyrins (Figure 2) have been considered a good alternative to the use of natural porphyrins due to their less complex structures and easy synthetic accessibility. The usefulness of this type of porphyrins can be improved through the adequate functionalization of β-pyrrolic positions or *meso*-aryl groups.

**Figure 3:**
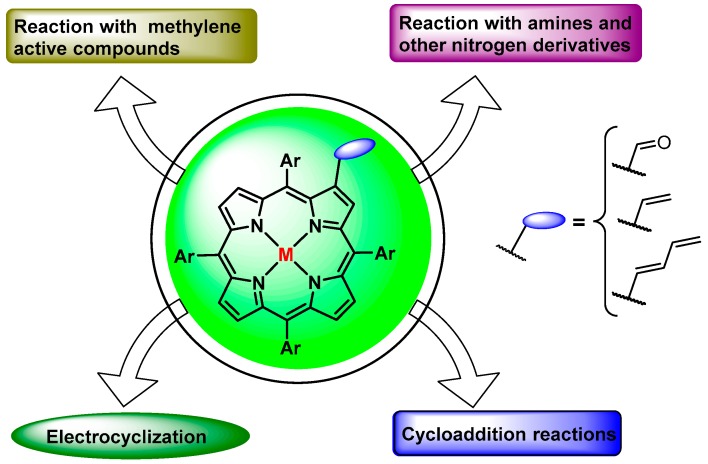
Types of reactions that are discussed in this review.

In this review the manipulation of formyl and vinyl groups located at the β-pyrrolic positions to access new *meso*-tetraarylporphyrins will be mainly highlighted. Formyl porphyrins are being used with success in the construction of more elaborate systems using several approaches such as the Horner-Emmons, Wittig, Grignard, McMurry, Schiff’s base, Knoevenagel and cycloaddition reactions [44,45,46,47]. The results reported here are mainly concerned with procedures involving reactions with methylene active compounds, amines and other nitrogen derivatives and with Wittig reagents. Further manipulation of some of the obtained derivatives, namely β-vinyl porphyrins, through pericyclic reactions will be also reported (Figure 3). However, when considered appropriate, similar transformations involving porphyrins with natural type substitution patterns will also be discussed.

Since symmetric *meso*-tetraarylporphyrins play a special role in these chemical modifications, synthetic strategies providing access to these templates will be succinctly discussed, followed by the synthetic strategies used to introduce the formyl and the vinyl groups at the β-pyrrolic positions. The numbering of the porphyrinic nucleus (from 1 to 24) will follow the IUPAC recommendations [48]. However, the interpyrrolic positions and the peripheral pyrrolic positions will also be referred to as *meso* and β-pyrrolic positions, respectively (Figure 2).

## 2. General Considerations about the Synthesis and Properties of *meso*-Tetraarylporphyrins

**Scheme 1:**
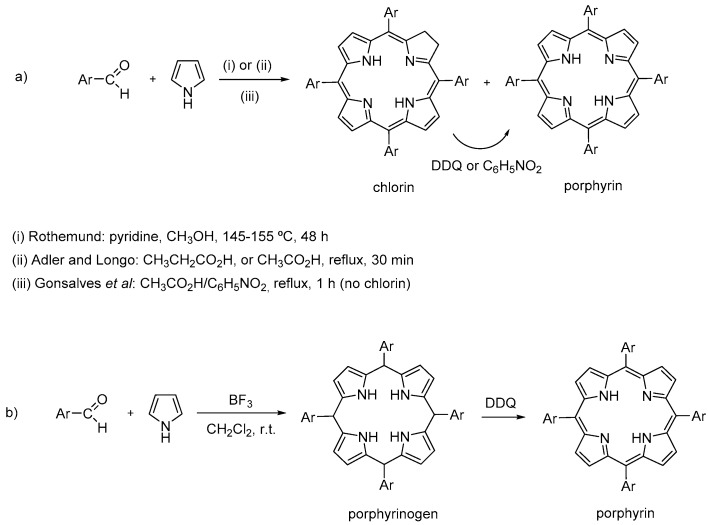
Conditions used to synthesize 5,10,15,20-tetrasubstituted porphyrins [60].

The experimental conditions affording access to symmetric free-base 5,10,15,20-tetraaryl-porphyrins are in general based on the Rothemund reaction reported in 1935 (Scheme 1a) [49,50,51]. The strategy described by Rothemund involved the condensation of aliphatic, aromatic and heterocyclic aldehydes with pyrrole using a mixture of pyridine and methanol, at 145–155 °C in a sealed tube under anaerobic conditions. Under these conditions a series of 5,10,15,20-tetrasubstituted porphyrins were obtained in low yields and contaminated with chlorin [52,53]. An improvement of this method that merits mention is the condensation under aerobic acidic conditions (propionic or acetic acid) described later by Adler and Longo. This variation allows one to obtain the corresponding porphyrins in higher yields [54], but even under these improved conditions the chlorin is still present and, in order to minimize it, Smith recommended the treatment of the reaction mixture with 2,3-dichloro-5,6-dicyanobenzoquinone (DDQ) [55]. Alternatively, as suggested by Gonsalves and co-workers [56], the condensation can be performed in a mixture of nitrobenzene and acetic acid. Under these conditions some porphyrins, namely 5,10,15,20-tetraphenylporphyrin (TPP, **1**), can be obtained directly from the reactional medium by simple crystallization.

A two-step strategy can be used for labile aldehydes that cannot survive the refluxing acidic conditions, as reported by Gonsalves [57] and Lindsey [58,59,60] in the late eighties for the preparation of 5,10,15,20-tetraalkylporphyrins and 5,10,15,20-tetraarylporphyrins, respectively (Scheme 1b). The condensation of pyrrole with the aldehyde is performed at room temperature in the absence of oxygen and light and using trifluoroacetic acid (TFA) or BF_3_ as catalyst, in order to maximize the formation of the porphyrinogen. The oxidative step can be performed with DDQ or *p*-chloranil and yields between 30% and 40% can be attained. An efficient access to several 5,10,15,20-tetraaryl-porphyrins (in yields ranging between 20% and 65%) by condensation of pyrrole and aryl aldehydes at room temperature and using equimolar amount of PCl_5_ or CF_3_SO_2_Cl in the presence of air as an oxidant was reported by Sharghi and Nejad [61,62].

The use of microwave irradiation (MW) can also be a good option for the preparation of 5,10,15,20-tetraarylporphyrins, as demonstrated by the number of reports and reviews on this topic [63,64,65,66,67]. In 2014, Pereira and co-workers demonstrated that some 5,10,15,20-tetraaryl- and tetraalkylporphyrins can be prepared in good yields in water (acts simultaneously as solvent and catalyst) under MW irradiation [68].

**Figure 4:**
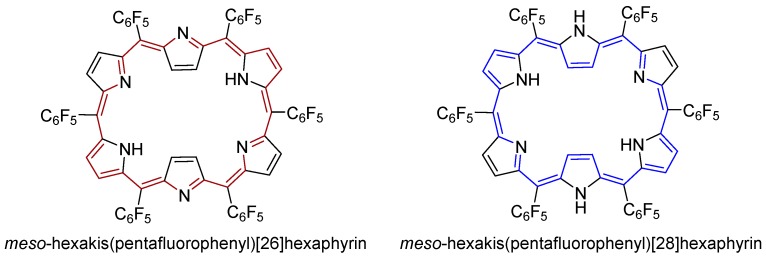
*meso*-Hexakis(pentafluorophenyl)hexaphyrins [69].

Depending on the experimental conditions, the Rothemund reaction can also give access to inverted and expanded porphyrins. In particular, we discovered that the condensation of pyrrole with pentafluorobenzaldehyde in acetic acid and nitrobenzene, gives the expected 5,10,15,20-tetrakis (pentafluorophenyl) porphyrin (Ar = C_6_F_5_) and also stable *meso*-hexakis(pentafluorophenyl)[26]hexaphyrin and -[28]hexaphyrin, respectively, with violet and blue colours (Figure 4) [69]. Based on their colours, it was a real surprise when we found from spectroscopic and X-ray single crystal diffraction data that these compounds have aromatic features. Since then, expanded porphyrinoids have emerged as a well-recognized group due to their unique redox and metal coordination behaviors, conformational flexibility and chemical reactivities, as excellently reviewed by Tanaka and Osuka [70].

Due to their aromatic character porphyrins can undergo, among other reactions, electrophilic aromatic substitutions. In *meso*-tetraarylporphyrins this type of reaction usually occurs at the more sterically accessible β-pyrrolic positions, although depending on the experimental conditions, selective transformations on the aryl groups (e.g., nitration, sulfonation) can also be performed [44]. Without losing their aromatic character (the aromatic delocalization only requires 18 of the 22 conjugated π-electrons) porphyrins can undergo other reaction types at the β-pyrrolic positions like attacks by nucleophiles, pericyclic reactions, oxidation and reduction reactions [44,71]. Porphyrins can also give reactions at the inner core positions, namely protonation, deprotonation, coordination with a wide range of metals, and *N*-alkylation [44]. As mentioned above, the main aim of this review is to show how simple formyl or vinyl groups, located at a β-pyrrolic position can lead to a wide range of compounds, sometimes unpredictably at a first glance.

## 3. β-Formylation of *meso*-Tetraarylporphyrins

One of the most efficient protocols to introduce a formyl group at the porphyrin periphery was based on the Vilsmeier-Haack reaction. The use of this reaction in the porphyrin field was reported for the first time by the groups of Inhoffen and Johnson in 1966 [72,73,74].

**Scheme 2:**
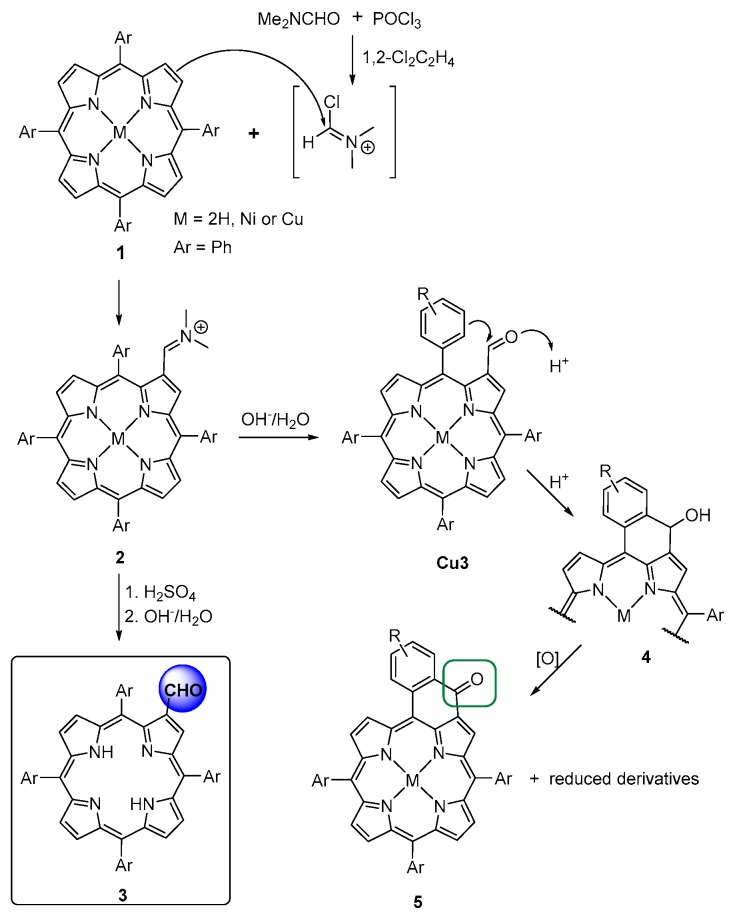
β-Formylation of *meso*-tetraarylporphyrins under Vilsmeier conditions [75,82,83].

The Vilsmeier–Haack formylation of *meso*-tetraarylporphyrins like TPP (**1**) at the β-positions are usually carried out by using the corresponding Ni(II) or Cu(II) complexes due to the compromise between electronegativity and acid lability they offer (Scheme 2) [75,76,77,78,79]. Under the acidic Vilsmeier conditions, the Zn(II) and Mg(II) complexes are demetallated and protonated and consequently the macrocycle reactivity is highly reduced for the β-formylation [80,81].

Attempts to demetallate the copper(II) complex of β-CHOTPP (Cu**3**) with strong acid led to the formation of the green naphthoketone **5** as a result of the acid-catalysed cyclization of the formyl group with the neighbouring *meso*-aryl group [82]. Callot confirmed that the green ketone is one of the products resulting from the dismutation of the intermediate allylic alcohol **4** (Scheme 2) [83]. Similar intracyclizations were reported by Dolphin and co-workers in their studies with *meso*-tetraarylporphyrins bearing *meso*-methoxyphenyl substituents [79].

Fortunately, Crossley and co-workers reported that the demetallation of the iminium salts Cu**2** with concentrated H_2_SO_4_ can afford, after basic hydrolysis, the corresponding free-bases **3** in good yield and on multi-gram scale (Scheme 2) [84]. Under these conditions the β-formyl-*meso*-tetraphenylporphyrin (**3**, β-CHOTPP) was isolated in an overall yield of 95%. A similar strategy, previously reported, but using the cobalt complex of TPP afforded the free-base **3** (Ar = Ph) in 65% yield [85].

**Scheme 3:**
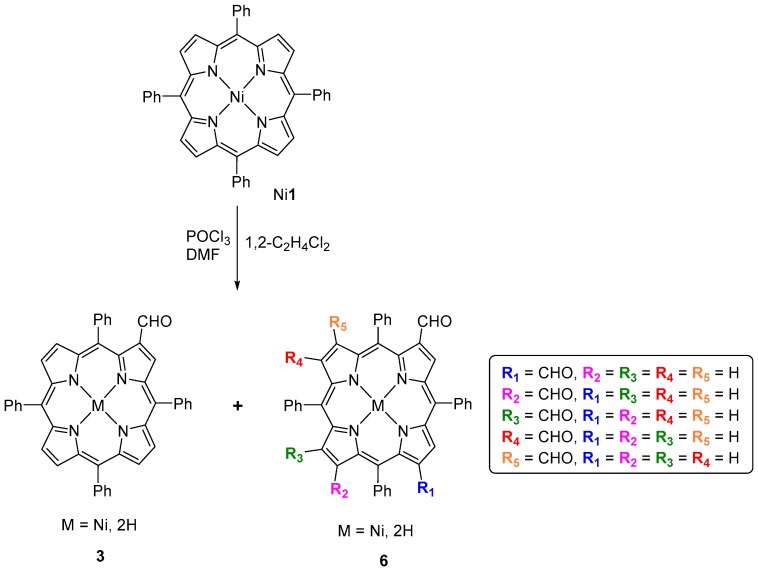
β,β′-Diformylation of *meso*-tetraphenylporphyrin [86].

β,β′-Diformyl-*meso*-tetraphenylporphyrins are also formed (in less than 4% yield) during the β-formylation of Ni(II)TPP (Ni**1**). Considering that these compounds could be potentially useful for further functionalization, and aiming at their syntheses, we decided to perform the Vilsmeier reaction in the presence of a larger excess of POCl_3_-DMF and using longer reaction times than employed in conventional conditions. Under these modified Vilsmeier conditions the diformyl compounds Ni**6** or the corresponding free-bases (as discussed above, the demetallation was performed at the iminium stage) were obtained in ca. 30% yield (Scheme 3). It was also verified that the direct formylation of the mono-β-formyl derivative Ni**3** can afford the five diformyl derivatives Ni**6** in 71% yield [86]. It was realized that the first introduced formyl group does not exert a substantial effect on the introduction of the second one.

Mironov and co-workers also reported the diformylation of the Ni(II) complex of *meso*-tetrakis(*p*-methoxyphenyl)porphyrin and of its chlorin [87,88]. Their studies agree with our previous observation about the effect of the first introduced formyl group in the subsequent formylation. Also, the relative positions of the two formyl groups have a strong effect on the spectroscopic characteristics of the porphyrin derivatives. A different situation was observed for the chlorin derivative. In this case the first formyl group goes to the pyrrole ring adjacent to the reduced unit and the subsequent formylation is preferably directed into the pyrrole opposite to the reduced unit. In that work [88] it was also mentioned the access to β,β′-dicyano-*meso*-tetrakis(*p*-methoxyphenyl) porphyrins by treating the corresponding β,β′-diformylporphyrins with hydroxylamine in pyridine followed by dehydration of the oximes with acetic anhydride.

The use of MW irradiation to prepare β-formyl porphyrins was reported by Yaseen et al. in 2009 [89]. The authors found that the acidic Duff conditions had to be used in alternative to the standard Vilsmeier–Haack method. The β-formylated products were obtained in 50–55% yields after irradiating the Zn(II) or Cu(II) complexes of the selected porphyrins in the presence of urotropine and acidified silica gel for a period of 18 min. However, Moura et al., demonstrated that it is possible to use microwave irradiation to formylate, under Vilsmeier-Haack conditions, the Ni(II) and Cu(II) complexes of TPP and of 5,10,15,20-tetraarylporphyrins with electron-donating or electron-withdrawing substituents (Table 1) [90]. The authors reported that it is possible to reduce the reaction times from ca. 18 h (conventional heating method) to 30 min or less under MW radiation, but keeping the efficiency of the process or even improving the yields obtained (Table 1). In the same study it was confirmed that the yields were not affected by reaction scale-up. Another significant advantage is the small volume of solvent required which is five-fold lower than the used in the classical heating conditions.

## 4. Reactivity of β-Formyl-*meso*-tetraarylporphyrins with Methylene Active Compounds

**Figure 5:**
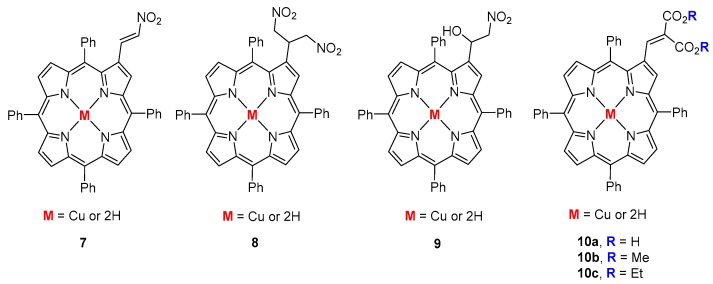
Products from the condensation of β-CHOTPP or its Cu(II) complex with nitromethane or malonic acid derivatives [91].

The condensation of formyl porphyrins with methylene active compounds has not only been used in simple modifications of the porphyrin periphery, but also in the construction of more elaborate systems [44,45]. For instance, Ponomarev and co-workers [91] found that the reaction of β-CHOTPP **3**, or of its copper(II) complex (Cu**3**), with nitromethane is dependent on the solvent used (Figure 5). When the reactions were performed in a mixture of acetic acid and butylamine the 2-(2-nitrovinyl)porphyrins **7** were isolated in moderate to excellent yields. However, when the reaction was carried out in a mixture of dimethylformamide (DMF) and butylamine the dinitro derivatives **8** were isolated (≥78% yield). When the reaction was carried out in liquid NH_3_ the nitroalcohols **9** were formed (≥90% yield). In the same publication it was also reported that the reaction of the Cu(II) complex of β-CHOTPP with malonic acid or with its methyl or ethyl esters led to the corresponding condensation products **10** in high yield (≥87%). The authors found that the demetallation of the copper complexes with POCl_3_ treated with water afforded the corresponding free-bases in excellent yields; it was highlighted that this protocol is a good alternative to the conventional treatment with concentrated H_2_SO_4_ which leads to a great decomposition of the porphyrin derivatives. Additionally, transformations of derivatives **10**, namely reduction of the double bond, saponification and decarboxylation, were also reported.

**Scheme 4:**
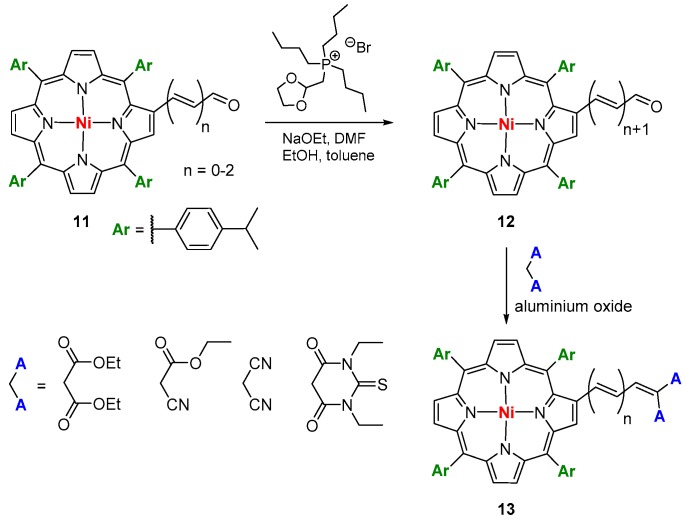
Reaction between β-formylporphyrins and acceptors [92].

The same type of strategy was used by Chen et al. to prepare the 2-substituted nickel *meso*-tetraarylporphyrins **13** (Scheme 4) [92,93]. These derivatives were prepared by reacting the appropriate Ni(II) β-formylporphyrin **12** with diethyl malonate, ethyl cyanoacetate, malononitrile, and *N*,*N′*-diethylthiobarbituric acid in the presence of aluminum oxide. The formylporphyrins **12** were obtained from *meso*-tetrakis(4-isopropylphenyl)porphyrins (**11**, *n* = 0) by repeating the Wittig oxyprenylation procedure on the shorter aldehyde homolog **11** (Scheme 4). The unexpected perturbed absorption spectra found for some of these substituted porphyrins (splitting of the Soret and Q bands) were justified by considering the stronger interaction between the porphyrin π-system and the electronic acceptor unit through the olefin linkage in addition to their reduced symmetry. It was commented that the unperturbed absorption spectra observed for many β-substituted *meso*-tetraphenylporphyrins can be justified by considering the weak electron accepting power of the substituent which can be an obstacle on the conjugation pathway or due to steric hindrance.

**Figure 6:**
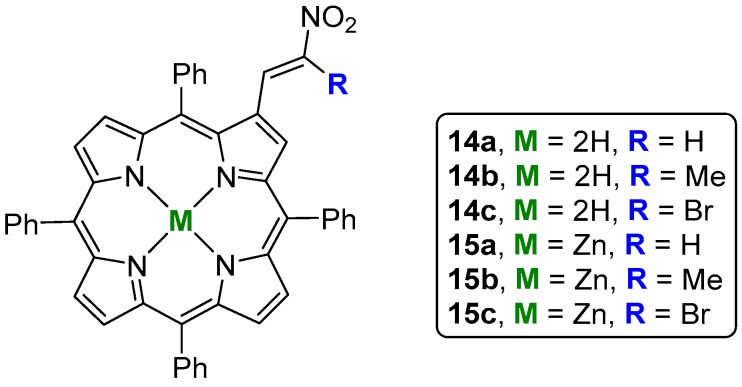
β-Nitroalkenyl-substituted *meso*-tetraphenylporphyrins [95].

Tandem mass spectrometry, namely electrospray tandem mass spectrometry (ESI-MS/MS) is a valuable tool for the rapid and sensitive analysis of porphyrins in mixtures and can also give important information about their structural characterization, namely of peripheral substituents [94]. This approach was considered to study the fragmentation pathways of β-nitroalkenyl-substituted *meso*-tetraphenylporphyrins **14a**–**c** and of their Zn(II) complexes **15a**–**c** (Figure 6), obtained by the classic condensation reaction between an aldehyde and a nitroalkane bearing α-hydrogen atoms [95]. In addition to the common fragmentation pathway of nitro derivatives, whose relative abundances are essentially controlled by the presence and absence of a metal and by the nature of the R substituent, it was found that losses of CH(NO_2_)R and HNO_2_ plus C_2_H_2_ are typical of free-base derivatives while the loss of OH, H_2_O, OH plus H_2_O and RCCH plus H_2_O were observed only for the complexes. It was also found that the nature of the R substituent affects the typical nitro group fragments. When CH_3_ was replaced by Br, the main loss of HNO_2_ changed to a combined elimination of the bromine atom with the typical nitro group fragments. Unusual fragmentations were also observed, namely the losses of RCNO, RCNO_2_ and HNO_2_ plus C_2_H_2_. This type of approach was explored by other groups to prepare dyads to be used in dye sensitized solar cells [96].

**Scheme 5:**
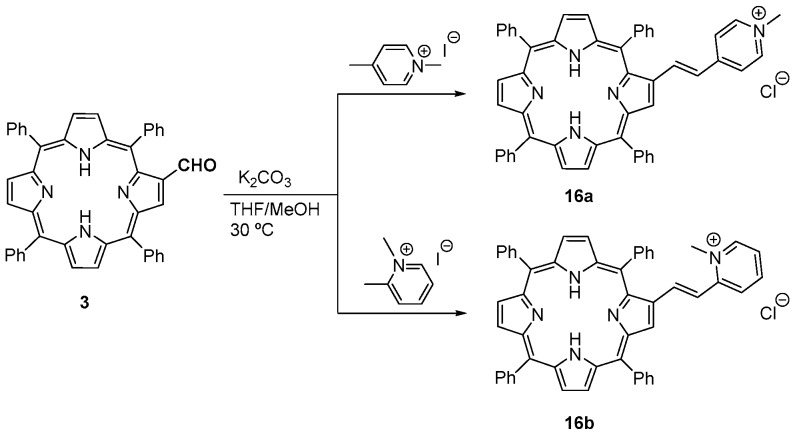
Aldol type condensation of β-CHOTPP **3** with 1,4-and 1,2-dimethylpyridinium iodides [97].

One of our first studies concerning the use of β-CHOTPP **3** in aldol type condensations was based on the simple concept that 2-and 4-alkylpyridines can undergo aldol-like condensations with carbonyl compounds upon treatment with strong bases, but the use of *N*-alkylpyridinium salts would allow the reaction to occur under milder conditions, as a consequence of the electron withdrawing effect exerted on the ring by the presence of the positive charge [97]. In fact, it was verified that the condensation of β-CHOTPP with 1,2-or 1,4-dimethylpyridinium iodide in the presence of K_2_CO_3_ affords the cationic β-vinyl substituted *meso*-tetraphenylporphyrin derivatives **16a**,**b** (Scheme 5). The synthesis of the neutral analogue of **16a** starting from **3** has previously been reported in the literature, but the procedure described required the reduction of the carbonyl function, followed by halogenation of the resulting hydroxymethyl group, its conversion into the corresponding triphenylphosphonium salt and then reaction with pyridine-4-carbaldehyde. This strategy affords both *E*-and *Z*-isomers, while our method leads to the *E*-isomer only [98,99,100].

The in vitro activity of derivatives **16a**,**b** against herpes simplex virus type I (HSV-1) was evaluated and although the two compounds displayed similar photocytotoxicity profiles, a striking difference was found in their photoinactivation efficiency. At the same concentration and after 5 min of irradiation, compound **16b** was able to photoinactivate 97% of the viral population, while compound **16a**, under the same conditions, displayed no virucidal effect [97].

**Figure 7:**
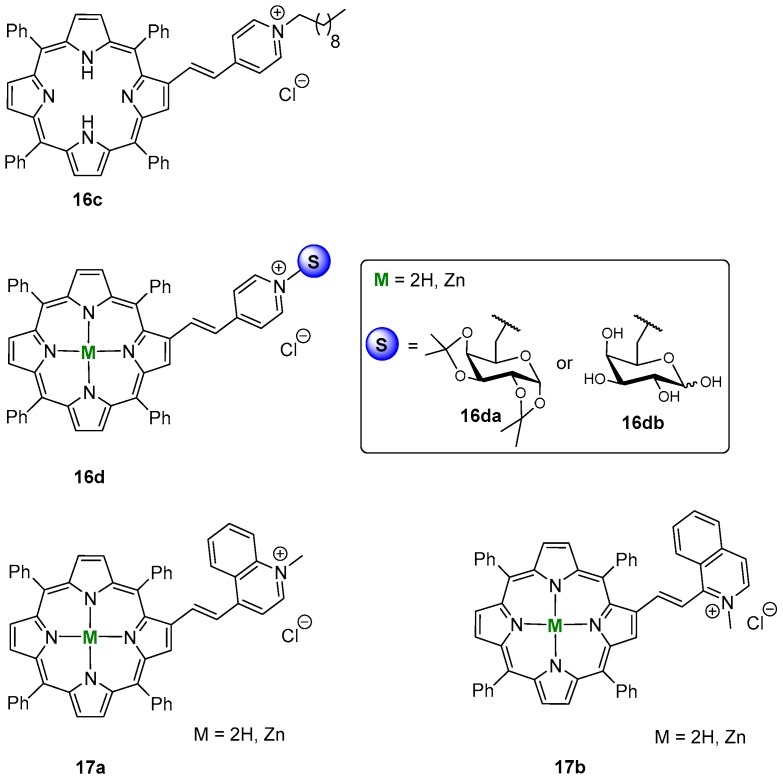
β-Vinylpyridinium- and β-vinylquinolinium-*meso*-tetraphenylporphyrin derivatives [103].

Interestingly, the two free-base isomers **16a** and **16b** were easily differentiated by ESI-MS and ESI-MS/MS studies [101]. Semi-empirical calculations allowed to conclude that the different electron density distribution was the responsible for the different gas phase behaviour of the two isomers and not the local distortion due to the presence of the β-vinylpyridyl substituents. It was also found that the different gas phase behaviour is less effective when the corresponding zinc(II) complexes were studied. The structural effects of the β-vinyl linker in the electronic properties of isomeric pyridinium porphyrins **16a** and **16b** were accessed later by steady state and transient spectroscopies both in organic solvents and in AOT reverse micelles, the biomimetic models of natural membranes [102]. Different contributions of the *trans* and *quinoid* resonance structures in the ground state were proposed for both isomers. Solvatochromic studies analysed for the Kamlet–Taft mutiparametric model showed that the solvatochromism of *para* isomer is ruled by solvent polarity, as expected for a charge transfer process in which an electron is transferred from the porphyrin to the pyridinium moiety, and being limited in the 2-substituted isomer **16b** due to steric hindrance to rotation. Nevertheless, a specific interaction with protic solvents was revealed by the characteristic emission spectra of the 4-substituted isomer **16a** in alcohols with increasing number of carbons. The spectroscopic behaviour of **16a** and **16b** isomers was also significantly different in AOT reverse micelles emphasizing the structural effect of the β-vinyl linker. While compound **16b**, the cytotoxic compound, resides at the interface toward the organic phase, compound **16a**, the non-cytotoxic one, does not interact with AOT reverse micelles. These different affinities towards membrane models may help to explain the higher virucidal effect of the 2-substituted isomer **16b**. As an extension of the previous synthetic strategy, another condensation involving β-CHOTPP **3** or Zn**3** with other *N*-alkyl derivatives of 2- and 4-methylpyridine and also of 2- and 4-methylquinoline gave rise to the cationic β-vinylpyridinium- and β-vinylquinolinium-*meso*-tetraphenylporphyrin derivatives **16c**,**d** and **17** (Figure 7) [103]. 

In this study it was verified that the condensations with 1,2- and 1,4-dimethylquinolinium iodides require slightly different conditions, namely being performed at 60 °C and using pyridine as solvent. The capability of this series of porphyrin derivatives to generate singlet oxygen was qualitatively estimated by measuring the absorption decay of 1,3-diphenylisobenzofuran (DPBF) and the results obtained showed that the best singlet oxygen producers were compounds **16b** and **16da** (with the sugar unit protected). Compounds with the quinolinium moiety did not produce singlet oxygen and the others showed an efficiency similar to TPP. The binding mode of the synthesized cationic derivatives with a short GC duplex oligonucleotide was evaluated by ESI-MS and the results indicated the probable occurrence of an outside binding. The UV-vis spectroscopy data pointed also to a non-intercalation process.

**Scheme 6:**
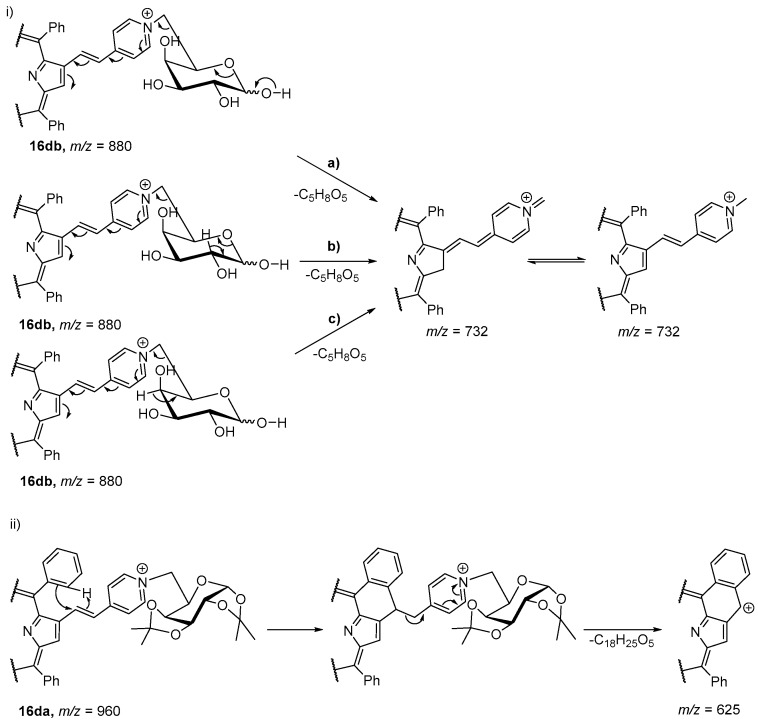
Atypical fragmentations detected in the ESI-MS/MS spectra of porphyrins **16d** [104].

The gas phase behavior of the cationic porphyrins **16d** bearing the galactose unit was compared by ESI-MS and ESI-MS/MS with the behavior of other glycoporphyrins bearing the same sugar unit linked directly to a pyridine or to an aminophenyl group located at a *meso*-position [104]. For all derivatives, the electrospray mass spectra of the monocharged porphyrins showed the expected M^+^ ions. It was also found from the fragmentation studies of these ions under ESI-MS/MS conditions that the elimination of the sugar residue as a radical is a common fragmentation pathway, but the loss of the sugar unit as a neutral fragment and cross-ring fragmentations typical of glyco-derivatives were only detected for the pyridinium glycoporphyrins, but not for ammonium glycoporphyrins. However, distinct fragmentations were observed for porphyrins **16d**. For instance, for derivative **16db** the major fragment in its MS/MS spectrum is due to loss of fragment C_5_H_8_O_5_ corresponding to 148 Da (base peak at *m*/*z* 732) which was explained by the cleavage of the C5–C6 bond of the sugar unit with the migration of one hydrogen from that unit to the porphyrin residue (Scheme 6i). For porphyrin **16da**, besides an abundant ion at *m*/*z* 732, due to the cleavage of the C5–C6 bond of the sugar unit as it was detected for the unprotected derivative **16db** (in here only pathways (b) and (c) are possible), it was observed an important ion at *m*/*z* 625. This ion, corresponding to the combined losses of the protected galactose unit and of pyridyl unit was justified by considering the typical intramolecular cyclization between the double bond and the *ortho* position of the adjacent phenyl ring (Scheme 6ii).

**Figure 8:**
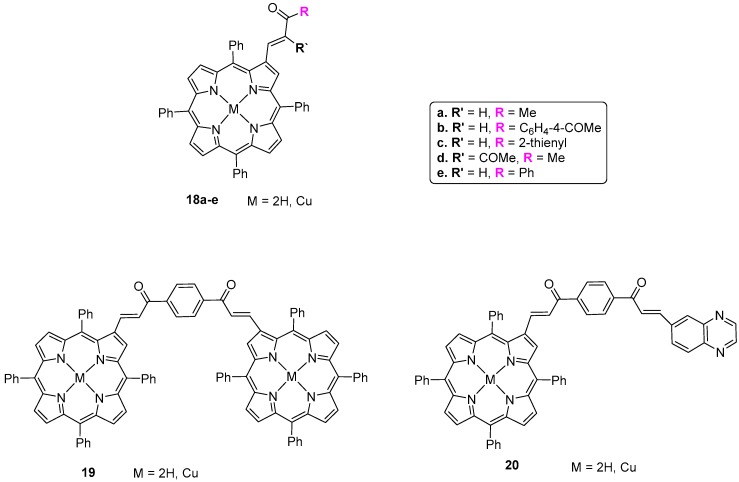
Structures of α,β-unsaturated derivatives obtained from the condensation of β-CHOTPP **3** with the adequate ketones [105,106].

The condensation of β-CHOTPP with methyl ketones has also been an important tool to functionalize the periphery of TPP giving access to new derivatives with potential applications in different fields. In 2003, Ishkov and co-workers reported the synthesis of porphyrin-chalcone-type derivatives **18** by reaction of β-CHOTPP or its Cu(II) complex with suitable ketones (acetone, 1,4-diacetylbenzene, 2-acetylthiophene, acetylacetone and acetophenone) in the presence of piperidine (Figure 8) [105,106]. These authors highlighted that the formation of the desired Claisen–Schmidt condensation products in appreciable amounts only occurs with the addition of a few drops of perchloric acid. The condensation of **18b** with β-CHOTPP or quinoxaline-6-carbaldehyde afforded the corresponding derivatives **19** and **20**. The authors emphasized that in all cases the free-base reacted at a higher rate than the copper(II) complex.

**Scheme 7:**
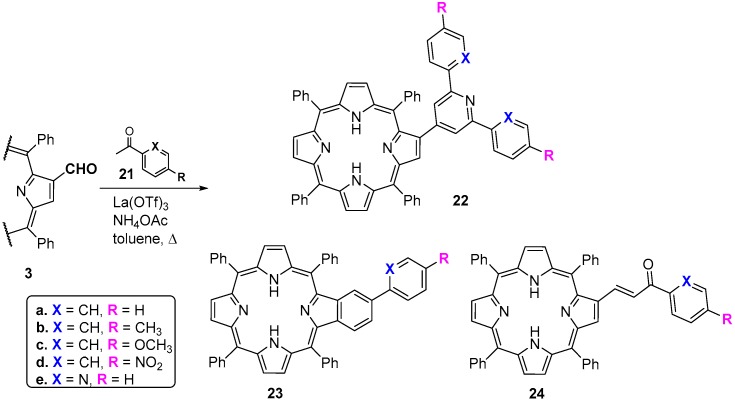
Condensation of β-CHOTPP with aryl methyl ketones **21** [107].

Moura et al. found that the condensation of β-CHOTPP **3** with aryl methyl ketones **21** in the presence of ammonium acetate and La(OTf)_3_ affords in just one step the Kröhnke-type porphyrin-2-ylpyridines **22** and the benzoporphyrins **23** in good yields (Scheme 7 and Table 2). 

Depending on the ketone reactivity, and the reaction time, the porphyrin–chalcone-type derivatives **24** were also isolated. These compounds were considered to be the key intermediates for the formation of **22** and **23** [107]. 

**Scheme 8:**
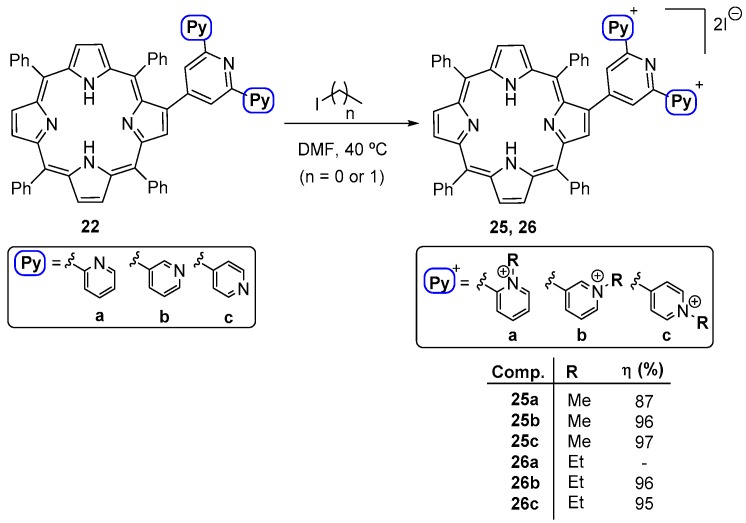
Cationization of porphyrin–terpyridine derivatives [108].

**Figure 9:**
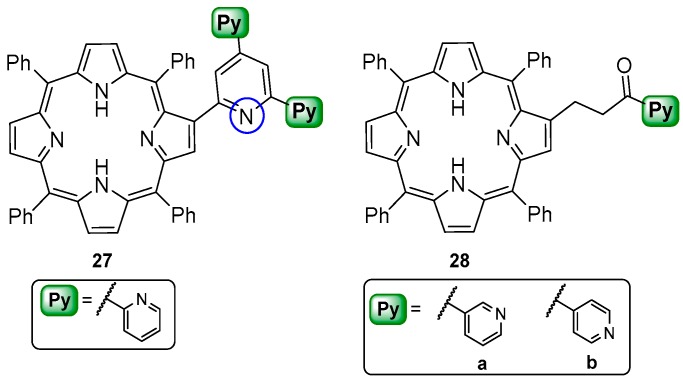
Other products obtained in the condensation of β-CHOTPP with acetylpyridines [108].

The Kröhnke-type approach was selected as the key step to obtain the cationic porphyrin-terpyridine derivatives **25** and **26** (Scheme 8) [108]. The condensation of β-CHOTPP **3** with the adequate acetylpyridines afforded after a carefully selection of the experimental conditions the required neutral derivatives **22a**, **22b** and **22c** as major products (yields between 45% to 48%) accompanied by the respective benzoporphyrins and porphyrin–chalcone-type derivatives. Interesting in this study, it was also isolated a new 2-(2,4-terpyridin-6-yl)-porphyrin **27** and the reduced compounds **28a** and **28b** (Figure 9). The quaternization of the pyridyl groups of **22a**, **22b** and **22c** was performed in the presence of the adequate alkyl iodide affording the dicationic derivatives in excellent yields.

The ESI-MS revealed to be a useful technique for the differentiation of these cationic derivatives through the formation of different diagnostic ions. The absence of the [M + H]^3+^ ion and the formation of the monocharged [M − CH_3_]^+^ ion allowed the differentiation of the *ortho* isomer **25a** from the isomers **25b**,**c**. On the other hand, formation of the [M + I^−^]^+^ adducts is a feature that is specific of the *meta* alkylated isomers **25b** and **26b**, enabling their differentiation from the *para* isomers **25c** and **26c**.

The efficiency of these dicationic derivatives to photoinactivate the bioluminescent *E. coli* was evaluated and the results show that their efficacy was dependent on the PS concentration, singlet oxygen efficiency, charge localisation in the porphyrin core and light irradiance [108]. A reduction in bacterial bioluminescence, up to 5.4 log, was obtained with the most efficient photosensitizer **25a**.

**Scheme 9:**
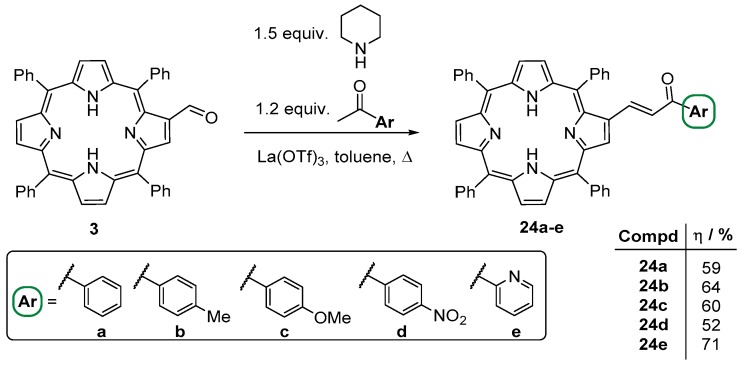
Condensation β-CHOTPP **3** with the aryl methyl ketones **21** in the presence of piperidine and La(OTF)_3_ [109].

In order to avoid the hazards associated to the use of perchloric acid in Claisen–Schmidt condensations reported by Ishkov et al. [105,106] Moura et al. re-examined the condensation of β-CHOTPP **3** with ketones **21** in the presence of piperidine and La(OTf)_3_, known as an excellent Lewis acid even in the presence of water (Scheme 9) [109]. The reactions performed in refluxing toluene afforded the expected porphyrin–chalcone-type compounds **24** in yields between 52% to 71%, accompanied by minor amounts of the corresponding benzoporphyrins (1–3%).

The sensing ability of these compounds towards a series of mono-, di- and trivalent metals was evaluated in solution and solid state by absorption and fluorescence spectroscopy and in the gas phase using MALDI-TOF-MS. The most significant changes in the ground and excited states were observed in the presence of several di- and trivalent metals, although compound **24b** was able to detect also the presence of Ag^+^. The results allowed to conclude that these probes can be analytically used to develop ratiometric molecular devices with high affinity and selectivity for Zn^2+^. Interestingly, from NMR titrations with Zn^2+^ it was possible to conclude that compounds **24b** and **24c** lost the internal *N*–H protons while in the interaction of the other derivatives **24a**, **24d** and **24e** the inner protons were maintained suggesting the formation of sitting-atop complexes as it was proposed by Fleischer [110,111]. The results showed also that these compounds when supported in polymethylmethacrylate (PMMA) films can detect and differentiate Zn^2+^ and Hg^2+^. Additionally, the results obtained by MALDI-TOF-MS showed the gas phase sensing abilities of the compounds towards Zn^2+^, Cu^2+^, Hg^2+^, Cd^2+^ and Ag^+^.

**Scheme 10:**
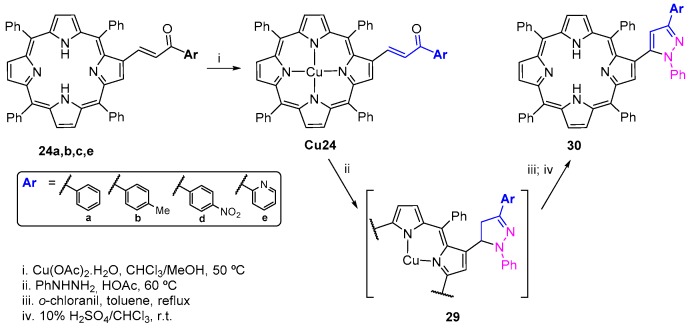
Reaction of porphyrin-chalcone derivatives **24** with phenylhydrazine [112].

Soon after, Moura et al. used the copper complexes of the β-porphyrin–chalcone derivatives **24a**,**b**,**d**,**e** to prepare the pyrazole–porphyrin conjugates **30** in excellent yields (up to 82%) (Scheme 10) [112]. It was reported that the best experimental conditions to obtain those compounds involve the reaction with phenylhydrazine in acetic acid at 60 °C followed by the oxidation of the porphyrin–pyrazoline derivatives **29** with *o*-chloranil and then demetallation with H_2_SO_4_ in CHCl_3_.

**Figure 10:**
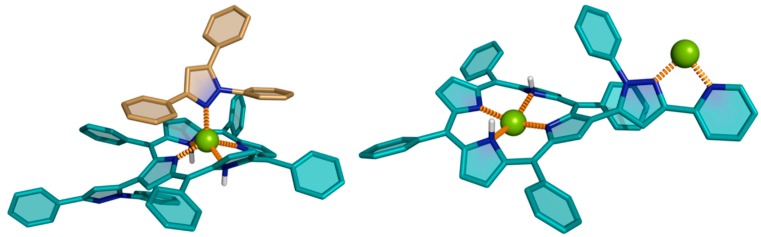
Views of the 1:2 (M/L) complex involving Zn^2+^ and **30a** and the 2:1 (M/L) complex involving **30e**, as calculated at the B3LYP/LanL2DZ level (adapted from [112]).

The sensing ability of these porphyrin–pyrazole derivatives to detect a series of mono, di and trivalent metal ions was also evaluated by absorption and fluorescence spectroscopies and the most significant changes in the ground and excited states were observed in the presence of Cu^2+^, Zn^2+^, Cd^2+^, and Hg^2+^. The results from the titrations of the conjugates with Zn^2+^ suggested complex formation in a stoichiometry of one metal ion per two ligands (M/L = 1:2), for ligands **30a**,**b**,**d**, and two metals per ligand (M/L = 2:1) for ligand **30e**; the lowest energy conformation for these two types of coordination determined through density functional theory (DFT) are shown in Figure 10. The same study showed that these probes have high selectivity towards Cu^2+^ and Ag^+^ in gas phase and when supported in poly (methyl methacrylate) towards Zn^2+^.

## 5. Reactivity of β-Formyl-*meso*-tetraarylporphyrins with Wittig Reagents

**Scheme 11:**
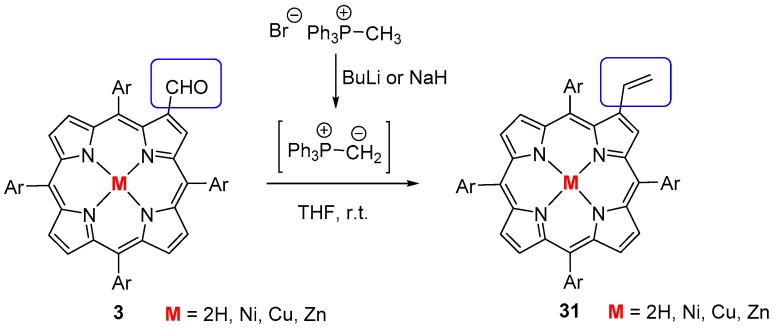
Synthesis of β-vinyl-*meso*-tetraarylporphyrins by Wittig reaction [75].

One efficient and simple procedure to introduce an alkenyl group at the porphyrin periphery of *meso*-tetraarylporphyrins involves the reaction of a β-formylporphyrin and a phosphorus ylide [44,85,113,114]. This approach was reported by Callot in 1973 [75] to prepare, for the first time, the β-vinylTPP **31**, an important template in our studies. In that pioneer report, the reaction was performed between the Ni(II) complex of β-CHOTPP **3** and the adequate ylide generated from methyltriphenylphosphonium bromide and BuLi in THF at room temperature. The Ni(II) complex of β-vinylTPP (Ni**31**) is obtained in 52% yield (Scheme 11). This protocol is also efficient when the free-base or the copper or zinc complexes are used; NaH is a good alternative to BuLi [75,115].

A complementary strategy was reported by Officer and co-workers that selected the porphyrin derivative as the phosphonium salt partner. The procedure, as mentioned above, requires the reduction of the carbonyl function, halogenation of the resulting hydroxymethyl group and its conversion into the corresponding triphenylphosphonium salt. The condensation with aldehydes afforded a series of TPP derivatives bearing vinyl substituents [98,99,100,116].

**Figure 11:**
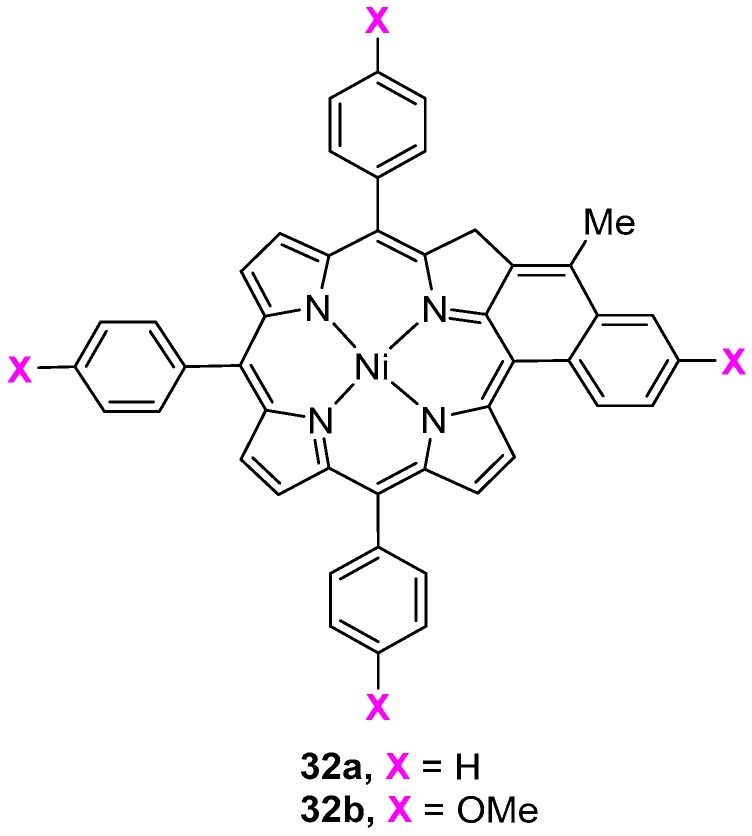
Naphthochlorin derivatives obtained from acid treatment of Ni(II) complex of β-vinylporphyrins **31** [117].

Interesting attempts to demetallate the Ni(II) complex of β-vinylporphyrins **31** (Ar = Ph or *p*-MeOC_6_H_4_) with strong acid conditions led to the isolation of the green naphthochlorins **32** (Figure 11) in excellent yields as a result of the acid catalysed intracyclization of the vinyl group with the neighbouring *meso*-aryl group [117].

**Scheme 12:**
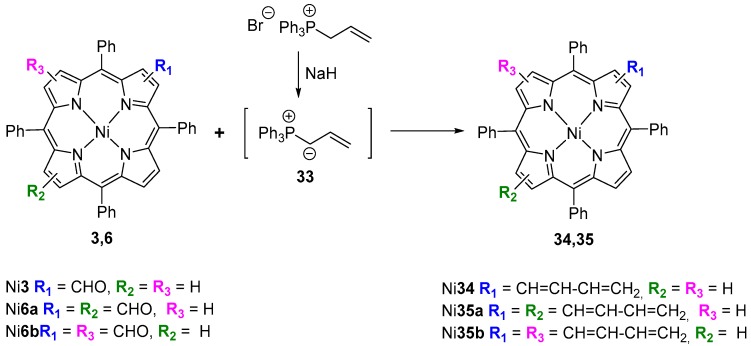
Synthesis of β-butadienyl- and β,β′-di(butadienyl)porphyrins [118].

Silva et al. showed that the Wittig reaction is also an excellent protocol to afford the β-butadienyl-**34** and β,β′-di(butadienyl)porphyrins **35** from the corresponding β-formyl and β,β′-diformyl derivatives and allylic phosphorus ylide **33** (Scheme 12) [118].

The reactivity of β-vinylporphyrins as dienes, dienophiles or dipolarophiles was also considered in our group as an important tool for further functionalization of *meso*-tetraarylporphyrins as shown below.

### 5.1. β-Vinylporphyrins as 4π Components in Cycloaddition Reactions

**Figure 12:**
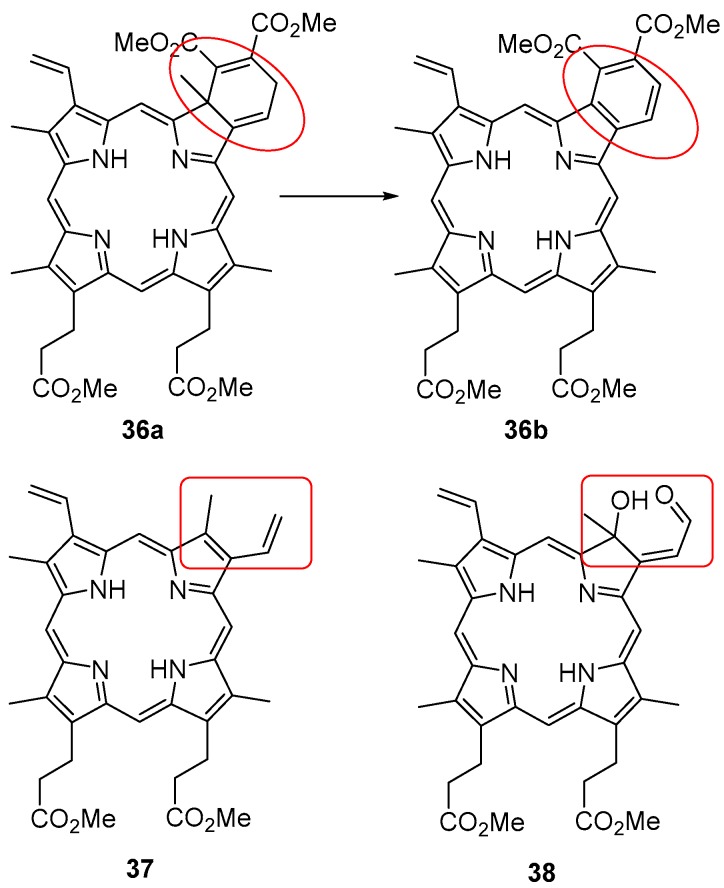
Protoporphyrin-IX dimethyl ester and adducts obtained in Diels-Alder reactions with singlet oxygen and dimethyl acetylenedicarboxylate (DMAD) [119,122,124,125].

Johnson and co-workers described for the first time the addition of activated dienophiles, such as dimethyl acetylenedicarboxylate (DMAD) or tetracyanoethylene (TCNE), to the β-β′ double bond of protoporphyrin-IX dimethyl ester through Diels–Alder reaction [119,120]. In the later decades, Dolphin and co-workers reported an intensive study concerning the use of protoporphyrin-IX dimethyl ester and analogues as dienes in the reactions with TCNE or DMAD. However, these reactions were revealed to be more complex than had been previously reported. In the studies with TCNE the formation of mono-and bis-adducts from [2 + 2] and [4 + 2] cycloadditions was observed and the expected isobacteriochlorin was only isolated in minor amounts [121,122,123,124]. In the presence of DMAD only monoadducts like **36a** were formed. These can be converted into the corresponding benzoporphyrins **36b** by loss of the methyl group (Figure 12) [122,124]. The possibility of β-vinylporphyrins to act as dienes was also suggested by Inhoffen et al. [125] to justify the formation of photoproducts of type **38** obtained (via a [4 + 2] intermediate) in the reaction of protoporphyrin-IX dimethyl ester **37** with singlet oxygen (Figure 12). It was mentioned that the reaction only occurs in one vinyl group and not in both.

**Scheme 13:**
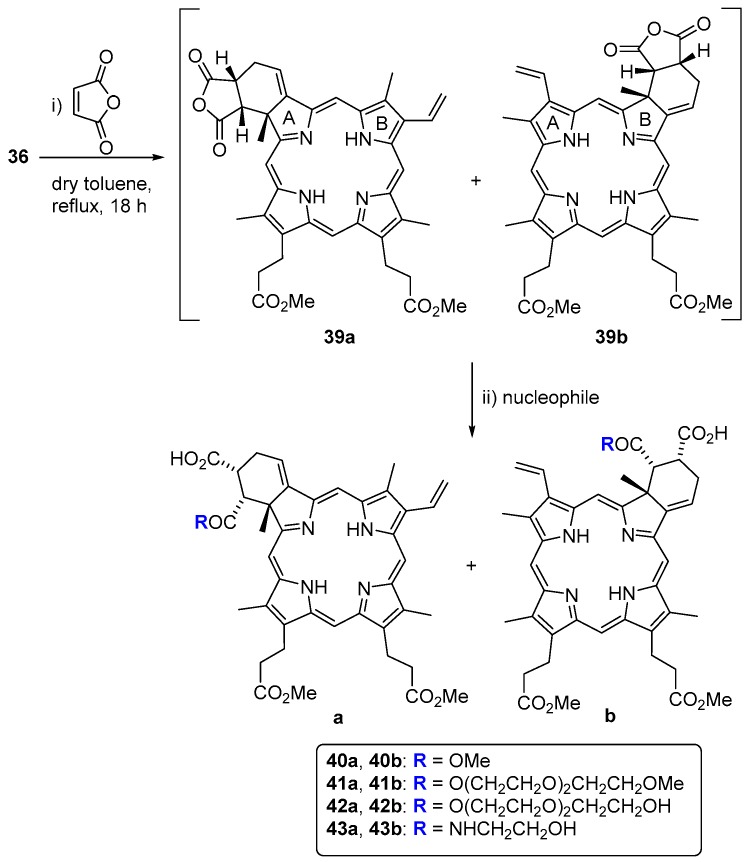
Synthesis of amphiphilic chlorin derivatives [126].

The Diels–Alder reaction between protoporphyrin-IX dimethyl ester and maleic anhydride was used for the synthesis of the amphiphilic chlorin derivatives **40**–**43** (Scheme 13) by ring-opening of the regioisomeric mono-adducts **39a** and **39b** with the adequate nucleophiles (alcohols or amines). The full characterization of the adducts **40**–**43** allowed to conclude that the final step in the process is highly regioselective and occurs at the carbonyl closer to the macrocycle. Additionally, the photophysical characterization showed that the compounds have adequate features for PDT applications [126,127].

**Figure 13:**
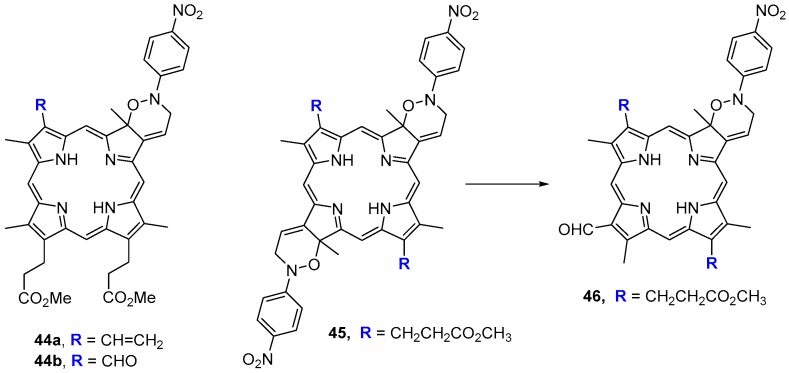
Adducts obtained in the reaction of protoporphyrin-IX and protoporphyrin-II dimethyl esters with *p*-nitronitrosobenzene [130].

The reaction of protoporphyrin-IX dimethyl ester with heterodienophiles was studied by us and other groups [63,128,129]. In particular, our studies were performed with nitrosobenzenes and only monoadducts such as **44a** were formed; the use of an excess of the dienophile conducted to the formation of the formyl-monoadducts of type **44b** (Figure 13). When the studies were extended to the centro-symmetric protoporphyrin-II dimethyl ester the chlorins and the expected bacteriochlorins **45** were identified by visible spectroscopy, NMR and MS; the bis-adducts (as a mixture of diastereoisomers) showed to be too unstable to support chromatography and gave rise to the formyl-monoadduct **46** [130].

**Scheme 14:**
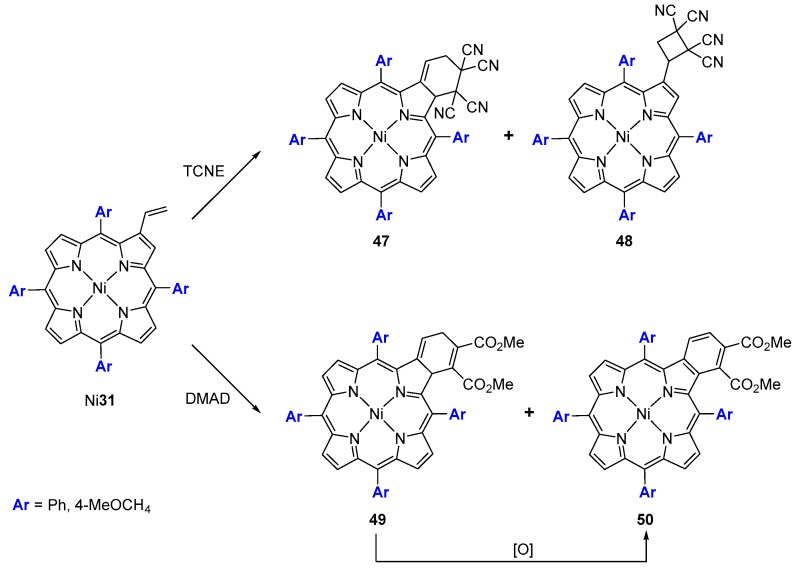
Reaction of the nickel(II) complex of β-vinylporphyrins with TCNE and DMAD [131,132].

The use of β-vinyl-*meso*-tetraarylporphyrins as dienes in Diels–Alder reaction was investigated for the first time in 1996 [131]. Faustino et al. showed that the reaction of Ni**31** with TCNE affords adducts **47** and **48** that result, respectively, from [4 + 2] and [2 + 2] cycloaddition processes (Scheme 14). The NMR studies showed that the formation of the [2 + 2] adduct can occur by rearrangement of the [4+2] chlorin. The reaction of β-vinylTPP Ni**31** (Ar = Ph) with DMAD afforded chlorin **49** and the benzoporphyrin **50**; the oxidation of **49** with DDQ afforded quantitatively the benzoporphyrin **50** [132]. Similar results were reported by Matsumoto et al. [133].

**Scheme 15:**
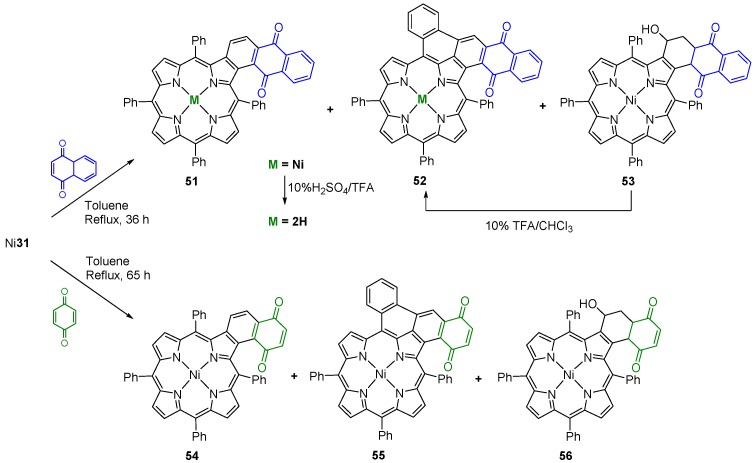
Reaction of the Ni(II) complex of β-vinylTPP Ni**31** with quinones [134].

Related reactions using 1,4-naphthoquinone and 1,4-benzoquinone as dienophiles did not afford the expected [4+2] chlorin adducts but the dehydrogenated derivatives **51** and **54** accompanied by a mixture of porphyrin–quinone derivatives with extended π-systems (compounds **52** and **55**, Scheme 15) [134]. The authors found that the hydroxylated compound **53** also isolated, is converted into porphyrin **52** (85% yield) after treatment with 10% trifluoroacetic acid and the demetallation of **52** with 10% sulfuric acid in trifluoroacetic acid afforded the corresponding free-base porphyrin **51** (M = 2H) in quantitative yield. Knowing that quinones are excellent agents in the dehydrogenation of hydroaromatic compounds, a plausible mechanism was proposed to justify the formation of the various products.

Matsumoto and co-workers [135] also reported the reaction of porphyrin Ni**31** with a range of dienophiles, including 1,4-naphthoquinone, and referred the formation of a single product in 76% yield that was identified as **51** (M = Ni).

**Scheme 16:**
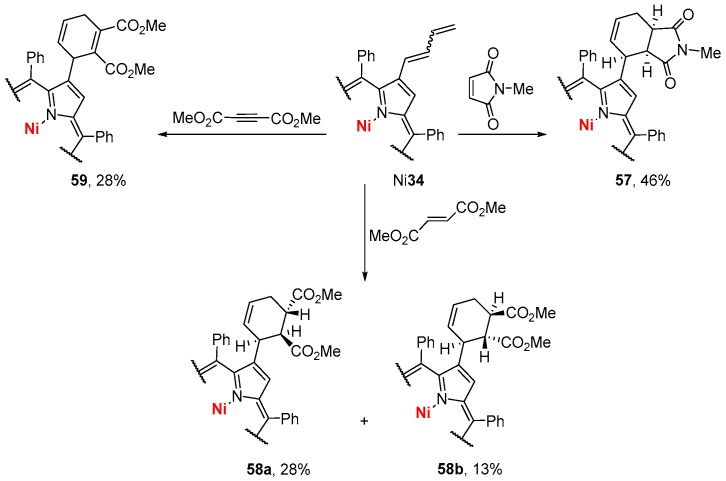
Reactions of butadienylporphyrin Ni**34** with activated dienophiles [118].

**Scheme 17:**
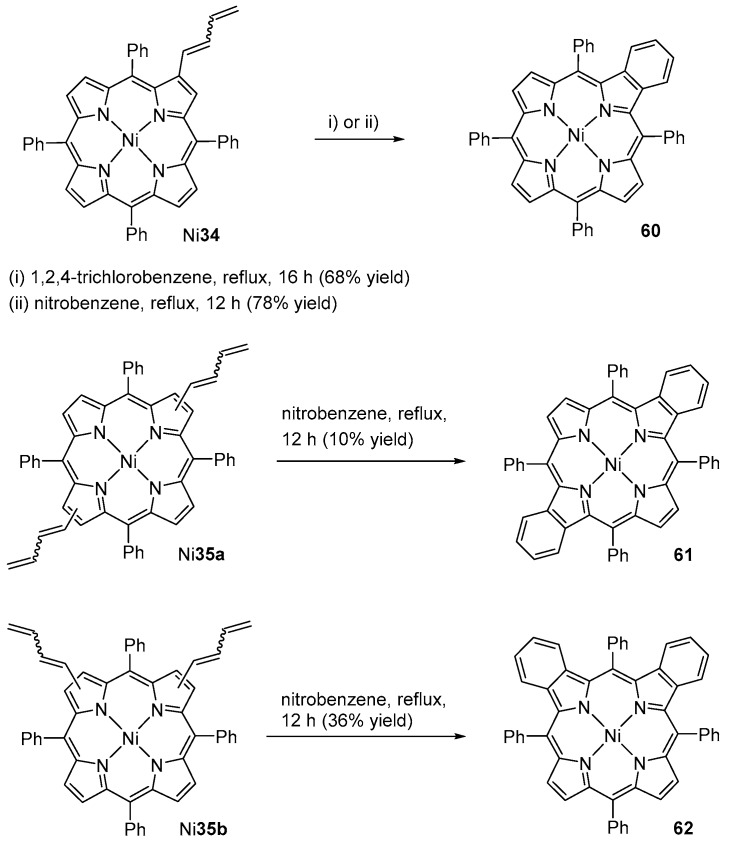
Electrocyclizations of β-butadienylporphyrin Ni**34** and β,β′-di(butadienyl)porphyrins Ni**35a**,**b** [118].

The reactivity of the β-butadienylporphyrin Ni**34** with *N*-methylmaleimide, dimethyl fumarate and DMAD was also evaluated (Scheme 16) [118]. The reactions were performed in the presence of an excess of the dienophile (6 equiv.) in refluxing toluene and afforded the expected adducts **57**–**59** in moderate yields. In the same article it was reported that, in the absence of a dienophile, compound Ni**34** undergoes electrocyclization followed by oxidation affording the benzoporphyrin **60** (Scheme 17). The extension of the studies to porphyrins Ni**35a** and Ni**35b** bearing two butadienyl groups in β-positions afforded respectively the opposite and adjacent dibenzoporphyrins **61** and **62** (Scheme 17). The authors found that the best solvent to perform these oxidative electrocyclizations is nitrobenzene. The single-crystal X-ray diffraction of the mono-and dibenzoporphyrin nickel complexes showed a strong distortion from the planarity of the porphyrin core [118].

### 5.2. β-Vinylporphyrins As 2π Component in Cycloaddition Reactions

**Scheme 18:**
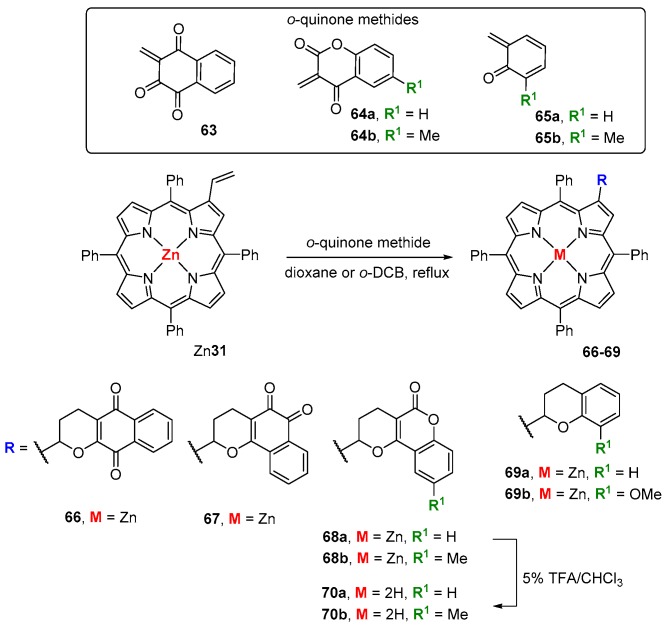
Reaction of Zn**31** with a series of *ortho*-quinone methides [136].

Porphyrins bearing a β-vinyl group can react as the dienophile in Diels–Alder reactions and as the dipolarophile in 1,3-dipolar cycloadditions. For example, the reactivity of the Zn(II) complex of β-vinylTPP (Zn**31**) was studied in the presence the *ortho*-quinone methides **63**–**65** generated in situ from the reaction of 2-hydroxy-1,4-naphthoquinone, 4-hydroxycoumarins or *o*-hydroxybenzyl alcohols with paraformaldehyde (Scheme 18). The reactions were performed in refluxing 1,4-dioxane or *o*-DCB until complete consumption of Zn**31** and the resulting dyads were isolated in 50% (**66**), 22% (**67**), 88% (**68a**), 95% (**68b**), 49% (**69a**) and 86% (**69b**) yields. The site selectivity and regioselectivity observed in these reactions are in agreement with the ones described for similar systems. The sensing ability of these Zn(II) conjugates towards different types of anions was evaluated by UV-vis and fluorescence measurements and alterations in both absorption and emission spectra or only in the emission spectra were detected in the presence of Cl^−^, CN^−^ and CH_3_COO^−^ [136].

The sensing ability of the free-base conjugates **70a** and **70b**, obtained in excellent yield by demetallation of the corresponding Zn(II) complexes with a mixture of TFA/CHCl_3_ (Scheme 18), was also evaluated but in the presence of different metal ions [137]. Both dyads showed a colorimetric effect (color change from purple to yellow) and an unprecedented selectivity for Hg(II), even in a mixture EtOH/H_2_O. The same colorimetric effect for Hg(II) was observed when the dyad **70a** was incorporated in a cellulose support material. The same derivative, both in solution and in the solid support, showed also a colorimetric effect at different pH values.

The coumarin-porphyrin dyad **68a** showed to be a potential probe towards the alkaloids caffeine, nicotine and cotinine, with a stoichiometry of one ligand per alkaloid [138]. Additionally, the interaction of **68a** with cotinine was verified by MALDI-TOF-MS and it was evidenced that this probe can detect small amounts of cotinine (2.5 ± 0.3 μM) in dam water samples.

**Figure 14:**
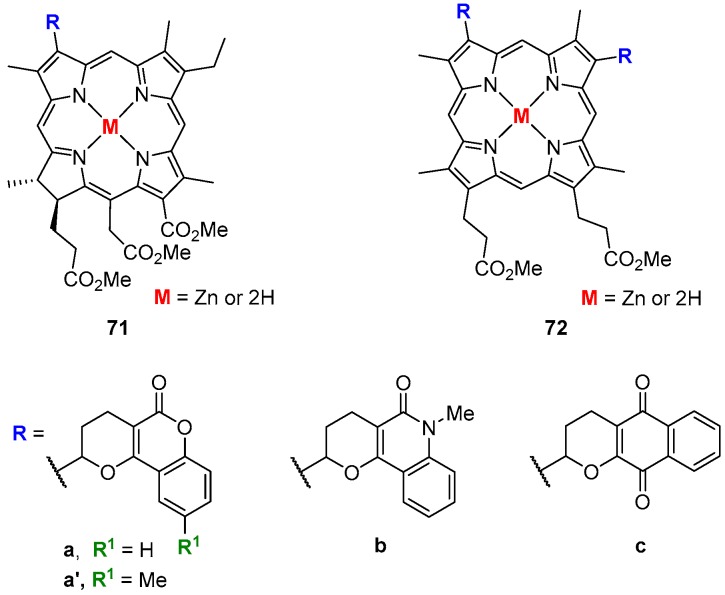
Compounds obtained from the reaction of natural-type porphyrin derivatives with *ortho*-quinone methides [139].

The extension of this hetero-Diels–Alder approach to the zinc(II) complexes of chlorin e_6_ trimethyl ester and protoporphyrin-IX as dienophiles and the *ortho*-quinone methides **63**, **64** and the ones generated from 4-hydroxy-*N*-methylquinolin-2-one and 2-hydroxy-1,4-naphthoquinone and paraformaldehyde, afforded the adequate dyads **71** and **72** in excellent yields (81–91%) (Figure 14) [139]. These complexes and the corresponding free-bases obtained from demetallation were isolated as mixtures of diastereomers and their abundances and characterization were determined by NMR. Aiming to find potential applications for these derivatives, their photophysical and electrochemical properties were evaluated. The results showed that the fluorescence quantum yield of the chlorin e_6_ dyads are higher than the ones of protoporphyrin-IX dyads. All dyads were able to generate singlet oxygen but the free-base chlorin e_6_ dyads showed an unexpectedly higher ability to generate singlet oxygen than the Zn(II) counterparts due to the higher tendency of the Zn(II) complexes to form aggregates. From the estimation of the intersystem crossing (ISC) quantum yield it was verified than the protoporphyrin-IX dyads have the highest ISC rates and that fact is in accordance with their higher singlet oxygen quantum yield when compared with the ones of the chlorin e_6_ series. From the electrochemical studies it was found that the peripheral modification of the ring affected the redox processes involved when compared with the starting templates.

**Figure 15:**
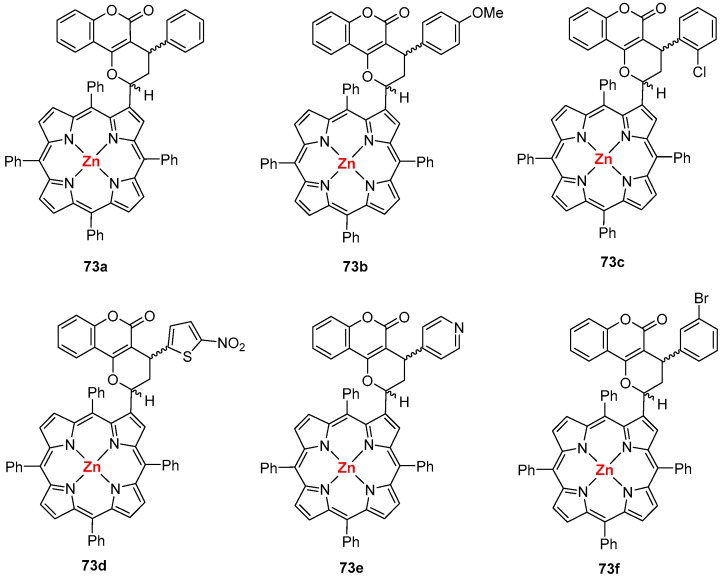
Dyads obtained from Zn**31** and various *ortho*-quinone methides [140].

The synthesis of six coumarin-porphyrin dyads (**73a**–**f**) (Figure 15) from porphyrin Zn**31** and *ortho*-quinone methides (generated in situ from 4-hydroxycoumarin and suitable aromatic aldehydes) was also reported and recently reviewed [140,141]. 

The reactions were carried out in different experimental conditions, namely in water under ohmic heating (a process in which the reaction mixture behaving as an electrical ohmic heater, is heated by passing an AC electrical current) [142]. The study allowed to conclude that the use of ohmic heating leads to a reduction of the reaction time, higher yields, selectivity and an easier work-up when compared with traditional organic solvents. Additionally, it was confirmed the potentiality of ohmic heating to perform reactions with complex systems like porphyrins.

**Scheme 19:**
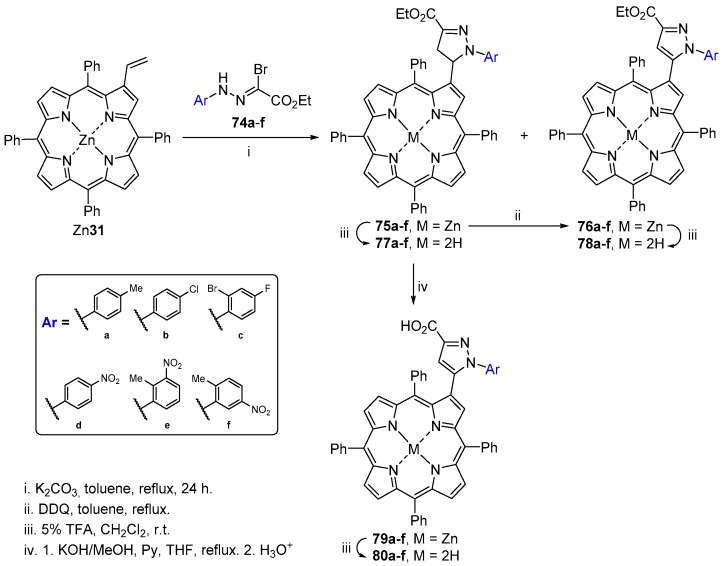
Reaction of Zn**31** with nitrile imines [143].

The reaction of Zn**31** with nitrile imines generated in situ from the ethyl hydrazono-α-bromoglyoxylates **74a**–**f** gave access to the corresponding pyrazolines **75** in good to excellent yields (63–92%) (Scheme 19) [143]. Treatment of pyrazoline adducts with DDQ affords the corresponding pyrazole derivatives **76** with moderate to excellent yields (50–96%). When the hydrolysis in the pyrazoline ester derivatives was attempted, it was observed the concomitant oxidation of the heterocyclic unit, giving rise to porphyrin–pyrazole derivatives bearing a carboxylic group (**79**) in very good yields. The free-bases **77**, **78** and **80** were obtained in excellent yields from the demetallation of the corresponding zinc(II) complexes with TFA. The photophysical properties of the pyrazoline- and pyrazole-porphyrin derivatives showed that the influence of the heterocyclic substituents is limited by the tendency of these molecules to aggregate. All other properties, and mainly the triplet kinetics, remain unaffected. The adducts with low tendency to aggregate showed very high singlet oxygen yield, and this makes these compounds interesting for their use as photosensitisers for PDT.

## 6. Reaction of β-Formyl-*meso*-tetraarylporphyrins with Amines and Other Nitrogen Derivatives

The reaction of β-formylporphyrins with amines or other nitrogen derivatives was successfully explored by us and others to functionalize the β-pyrrolic positions of *meso*-tetraarylporphyrins [44,63].

**Scheme 20:**
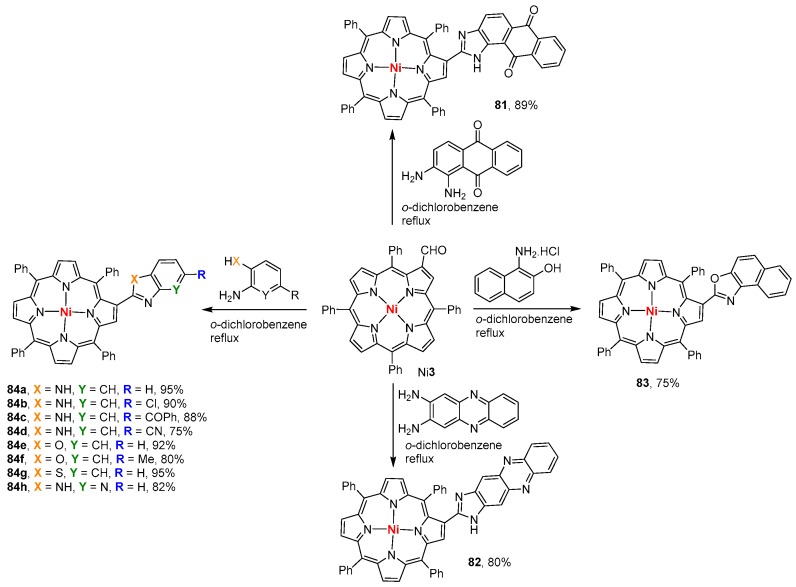
Reaction of β-CHOTPP with *o*-arylenediamines, *o*-aminophenols, *o*-aminothiophenol [144].

**Figure 16:**
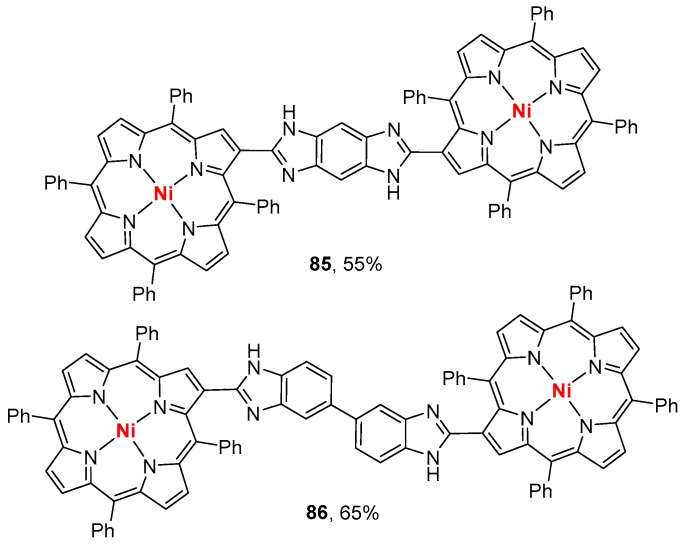
Dyads obtained from reaction of β-CHOTPP with benzene-1,2,4,5-tetraamine tetrahydrochloride and 3,3′-diminobenzidine [144].

Sharma and Nath reported that the condensation of the Ni(II) complex of β-CHOTPP with the adequate *o*-arylenediamines, *o*-aminophenols, *o*-aminothiophenol and 1-amino-2-naphthol in refluxing 1,2-dichlorobenzene gives access to the nickel(II) 2-benzazolo-5,10,15,20-tetraphenyl-porphyrins **81**–**84** in yields ranging from 75% to 95% (Scheme 20) [144]. A similar approach was used to synthesize the bis-benzimidazoloporphyrins **85** and **86** in 55% and 65% yields by reacting the Ni(II) complex of β-CHOTPP respectively with benzene-1,2,4,5-tetraamine tetrahydrochloride and 3,3′-diaminobenzidine (Figure 16). The authors commented that these reactions seem to proceed through the initial formation of the iminoporphyrin intermediate, which is followed by an intramolecular oxidative cyclization in the presence of air.

**Scheme 21:**
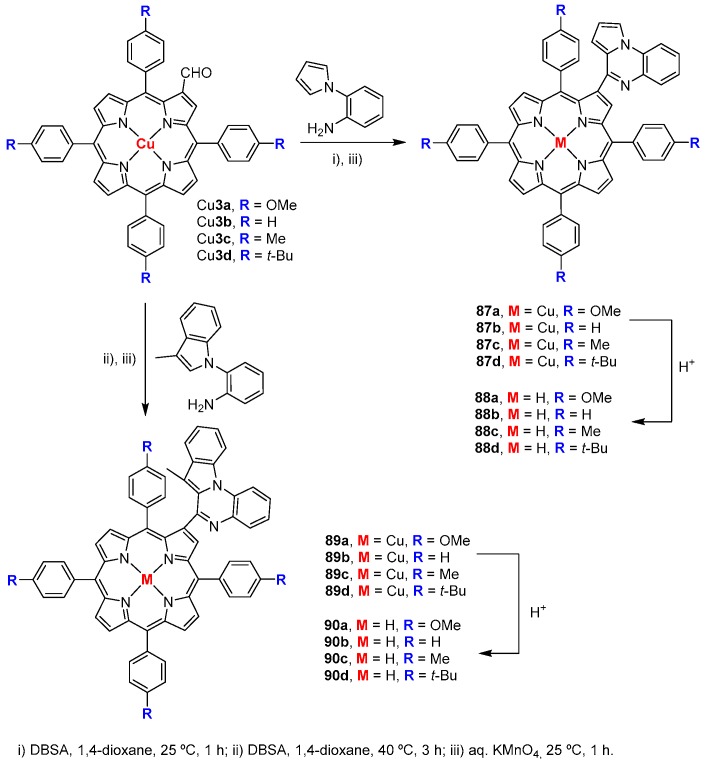
Structures of pyrrolo- and indolo[1,2-*a*]quinoxalinoporphyrins [145].

The same group reported recently a convenient access to the β-substituted copper(II) pyrrolo- and indolo[1,2-*a*]quinoxalinoporphyrins **87** (M = Cu, 83–89%) and **89** (M = Cu, 62–65%) through the condensation of the adequate copper(II) β-formyl-5,10,15,20-tetraarylporphyrins with 1-(2-aminophenyl)pyrrole or 2-(3-methylindol-1-yl)phenylamine (Scheme 21) [145]. The authors found that the best conditions for these Pictet–Spengler reactions is to perform the condensations in 1,4-dioxane at temperatures between 25 to 40 °C, in the presence of *p*-dodecylbenzenesulfonic acid, followed by in-situ oxidation with potassium permanganate at room temperature. Using conventional protocols an efficient access to the corresponding free-bases and to the Zn(II) and Ni(II) complexes were also reported [145]. The red shift observed in the electronic absorption and in some emission spectra of the pyrrolo- and indolo[1,2-*a*]quinoxalinoporphyrins in comparison to the corresponding *meso*-tetraphenylporphyrins were justified as being due to the extended π-conjugation.

**Scheme 22:**
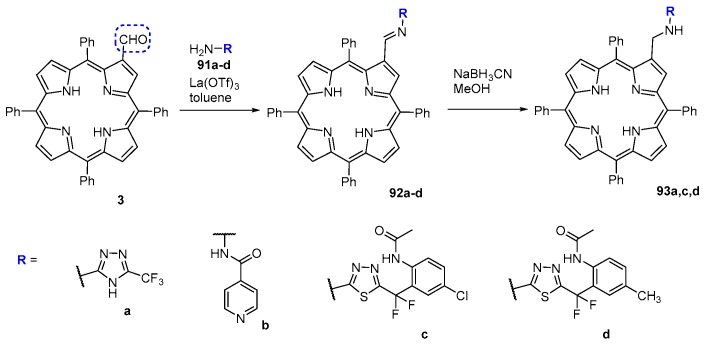
Reaction of β-CHOTPP with cutaneous leishmaniasis agents [147].

**Scheme 23:**
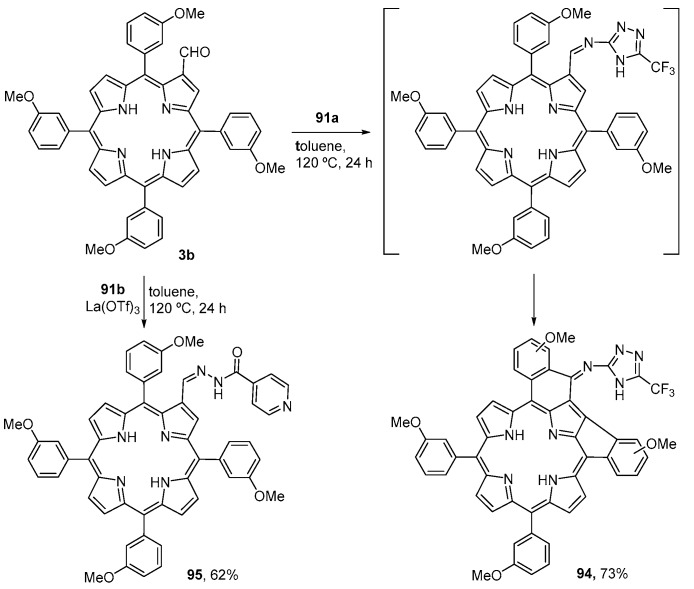
Reaction of β-CHO-*m*-MeOTPP with aminotriazole **91a** and isoniazid [147].

Giribabu and co-workers used the condensation between 1,2-diaminoanthraquinone and β-CHOTPP or its Zn(II) complex to prepare molecular bis(porphyrin)-anthraquinone triads having azomethine bridges [146]. The same approach was used to couple the cutaneous leishmaniasis agents aminotriazole **91a**, isoniazid (pyridine-4-carbohydrazide, **91b**) and the aminothiadiazoles **91c** and **91d** with the β-formylporphyrins **3** and **3a** (Ar = Ph and *m*-MeOC_6_H_4_) as a strategy to obtain molecules with dual function potentialities (Scheme 22 and Scheme 23) [147]. The reactions performed in the presence of La(OTf)_3_ afforded the expected imines **92a**–**d**, but due to their instability to chromatographic purification, they were reduced to the corresponding amines **93**; only imine **92b** could be isolated in 88% yield (Scheme 22). The study showed that the reactivity of β-CHO-*m*-MeOTPP (**3b**) with those aminoheterocycles is more complex under the previous conditions (Scheme 23). Curiously, the reaction with **91a**, but without La(OTf)_3_, afforded the double cyclized imine derivative **94** in 73% yield and the reaction with **91b** afforded the stable imine **95** (Scheme 23).

The singlet oxygen generation studies showed that all β-substituted porphyrin derivatives are able to generate singlet oxygen but derivative **94** is the most efficient one. Additionally the preliminary leishmanicidal properties obtained by molecular modelling and docking calculations showed that all synthesized compounds have higher values of relative affinity to the leishmanial arginase than current drugs.

**Scheme 24:**
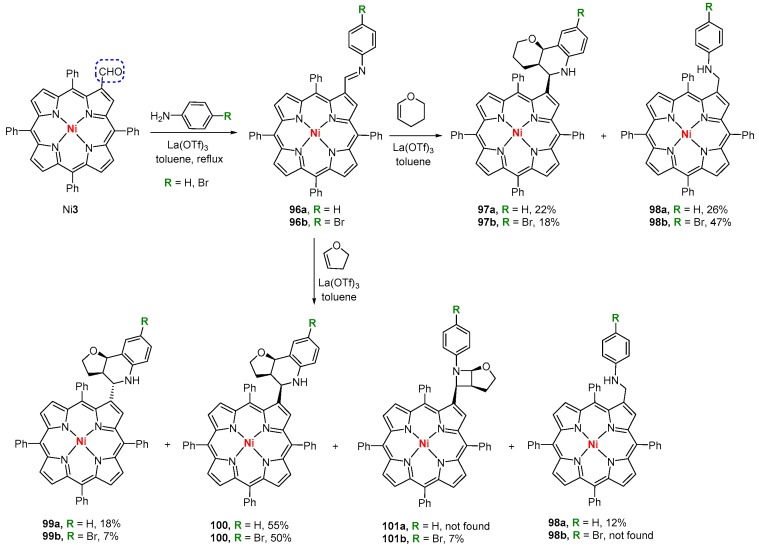
Reaction of β-iminoporphyrins with electron-rich dienophiles [148].

We also reported the reaction of the nickel(II) complex of β-CHOTPP (Ni**3**) with aniline and *p*-bromoaniline to obtain the β-iminoporphyrins **96** (Scheme 24) [148]. These imines react with electron-rich dienophiles such as 3,4-dihydro-2*H*-pyran and 2,3-dihydrofuran at room temperature and in the presence of La(OTf)_3_ to give the expected β-(tetrahydroquinoline)porphyrins **97**, **99** and **100** accompanied by the β-(arylaminomethyl)porphyrins **98**. When the reaction was performed with the β-iminoporphyrins **96b** and 2,3-dihydrofuran it was also observed the formation of the unexpected azetidine **101b**. The reduction of the imine bond requires the presence of both the dienophile and the catalyst.

**Scheme 25:**
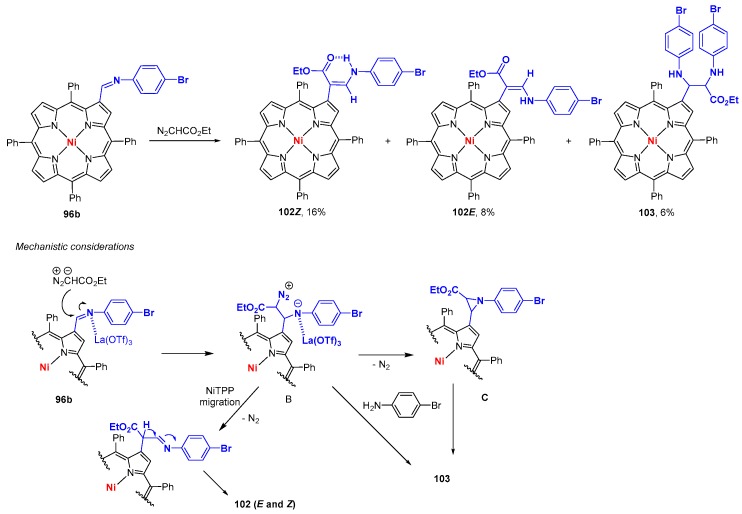
Reaction of β-iminoporphyrin **96b** with ethyl diazoacetate and mechanistic considerations [149].

Further studies showed that the β-iminoporphyrin derivative **96b** can react with ethyl diazoacetate in the presence of catalytic amounts of La(OTf)_3_ affording a mixture of the isomeric *E*/*Z* β-amino-α,β-unsaturated esters **102** and the α,β-diamino ester **103** (Scheme 25) [149]. The probable pathway proposed to justify the formation of the isolated compounds was based on the mechanistic data found in the literature about the aziridation of imines with ethyl diazoacetate catalysed by Lewis acids.

**Scheme 26:**
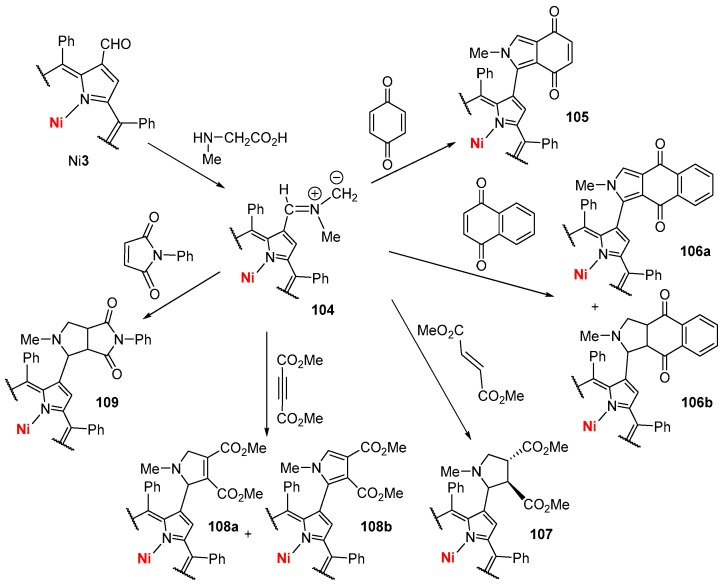
Reaction of the ylide generated from Ni**3** and *N*-methylglycine with various dipolarophiles [151].

The nickel(II) complex of β-CHOTPP (Ni**3**) reacts with *N*-methylglycine in refluxing toluene leading to the azomethine ylide **104** which can be trapped with dipolarophiles. Under this context, the reaction of **104** with fullerene was reported by Boyd and co-workers [150]. A related work, but involving other dipolarophiles, namely quinone, naphthoquinone, dimethyl fumarate, DMAD and *N*-phenylmaleimide was posteriorly described by our group (Scheme 26) [151]. The expected adducts (or their dehydrogenated derivatives) **105**–**109** were obtained in good yields.

**Scheme 27:**
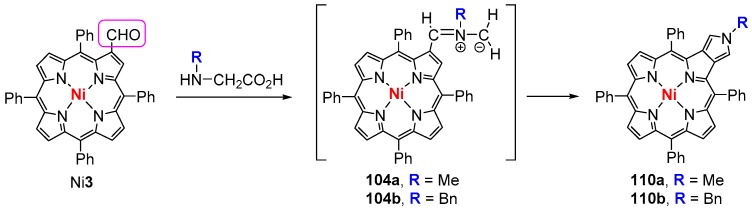
1,5-Electrocyclic ring closure of azomethine ylides to pyrrolo[3,4-*b*]porphyrins [152].

Interestingly, during these studies it was found that the porphyrinic azomethine ylides **104a**,**b** in the presence of a poor reactive dipolarophile or in its absence, undergoes 1,5-electrocyclic ring closure affording pyrrolo[3,4-*b*]porphyrins **110** in good yield (Scheme 27) [152]. This synthetic strategy to pyrrolo[3,4-*b*]porphyrins **110** is a good alternative to the one reported by Smith and co-workers to obtain similar compounds [153].

**Scheme 28:**
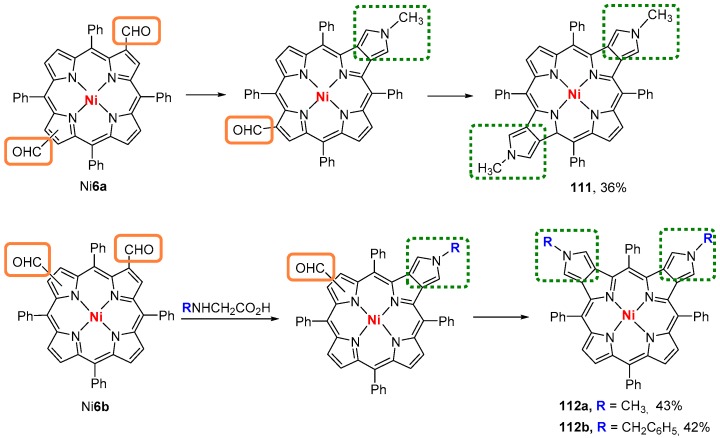
1,5-electrocyclic ring closure of azomethine ylides generated from the β,β′-diformyl derivatives Ni**6** [152].

The azomethine ylides generated from β,β′-diformylporphyrins **Ni6a**,**b** and *N*-methyl or *N*-benzylglycine also give 1,5-electrocyclic ring closure reactions affording the dipyrrolo[3,4-*b*:3,4-*l*]porphyrins **111** or dipyrrolo[3,4-*b*:3,4-*g*]porphyrins **112** depending on the relative positions of the two formyl groups (Scheme 28) [152].

**Scheme 29:**
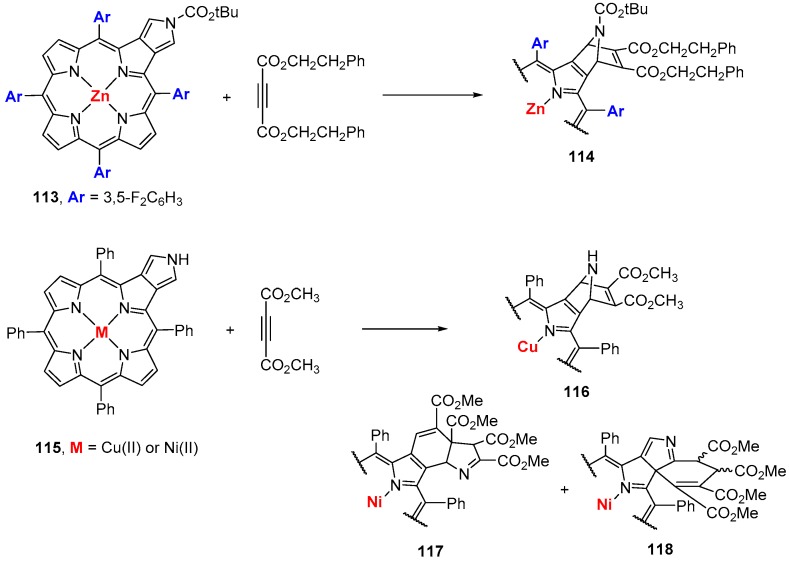
Reaction of pyrrolo [3,4-*b*] porphyrins **115** with acetylenedicarboxylates [154,155,156].

The reactivity of the exocyclic pyrrole unit in the pyrrolo[3,4-*b*]porphyrins **113** [154] and **115** [155] as diene was evaluated, respectively, by Knapp and Smith groups (Scheme 29). The studies were performed in the presence of acetylenedicarboxylates and afforded the expected adducts **114** and **116**. Further studies by Smith and co-workers showed that the pyrroloporphyrin **115** (M = Ni), in the presence of excess DMAD, reacts through a Diels–Alder reaction and a Michael addition affording the bis-adducts **117** and **118**. These adducts are converted into monobenzoporphyrins upon prolonged heating [156].

**Scheme 30:**
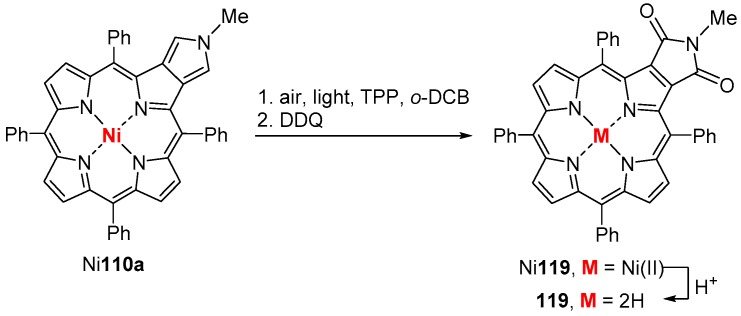
Reaction of *N*-methylpyrrolo[3,4-*b*]porphyrin Ni**110a** with singlet oxygen [157].

It was found that the easily accessible *N*-methylpyrrolo[3,4-*b*]porphyrin Ni**110a** can also react as diene with singlet oxygen affording the 1,3-dioxopyrrolo[3,4-*b*]porphyrin Ni**119** in 70% yield (Scheme 30) [157].

**Scheme 31:**
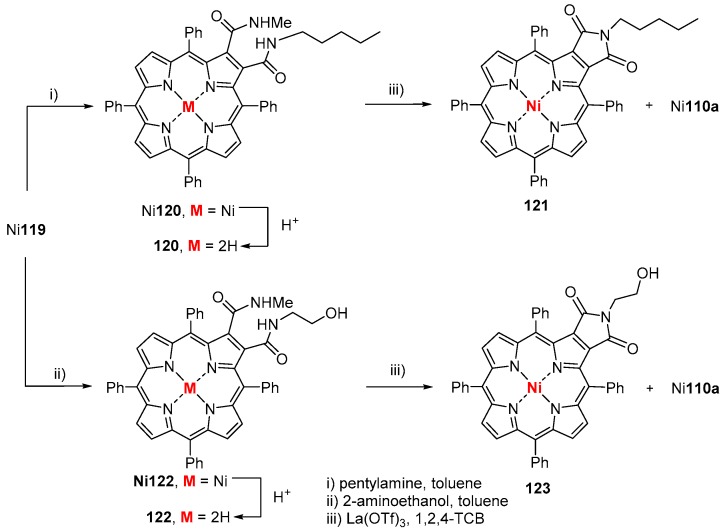
Synthetic access to other 1,3-dioxopyrrolo[3,4-*b*]porphyrins from Ni**119** [157].

The new imide was used as a template to obtain other 1,3-dioxopyrrolo[3,4-*b*]porphyrins **121** and **123** by reaction, respectively, with pentylamine and 2-aminoethanol, followed by ring-closure of the corresponding open counterparts Ni**120** and Ni**122** (Scheme 31) [157]. The UV-vis absorption spectra of the new compounds show significant red-shifts when compared with those of the nonsubstituted analogues. The ring-opening of imide Ni**119** in the presence of KOH afforded the corresponding 3-(methylcarbamoyl)-2-carboxylic acid in 84% yield. When this compound was heated in refluxing DMSO a decarboxylation occurred affording the corresponding amide derivative in 52% yield.

**Scheme 32:**
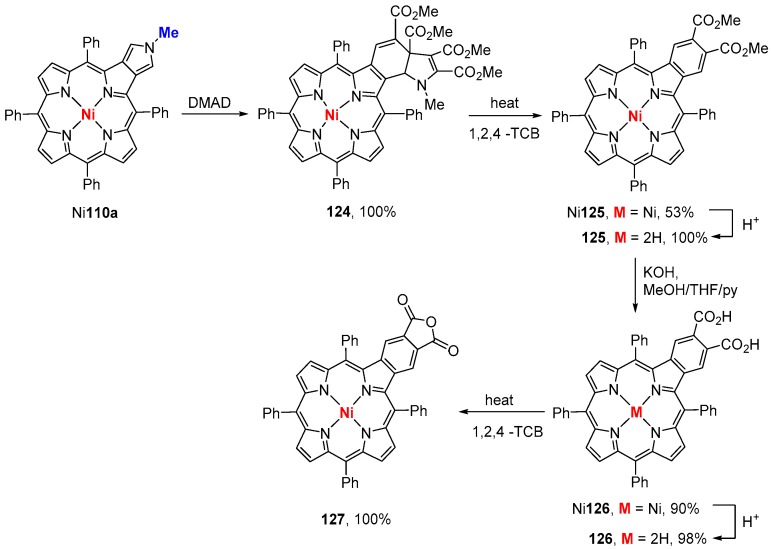
Synthetic access to anhydride **127** from pyrroloporphyrin Ni**110a** [158].

A protocol to convert the pyrrolo[3,4-*b*]porphyrin Ni**110a** into the corresponding benzoporphyrin-2^2^,2^3^-dicarboxylic anhydride **127** was also developed (Scheme 32) [158]. The first step involved the reaction of Ni**110a** with an excess of DMAD in refluxing toluene for 1 h, affording the bisadduct **124**, which after thermal decomposition in refluxing 1,2,4-TCB originated the benzoporphyrin-2^2^,2^3^-dicarboxylic ester **125** (53% yield). The hydrolysis of the ester groups to the “phthalic” acid **126** (90% yield) followed by dehydration in refluxing 1,2,4-trichlorobenzene afforded the anhydride **127**.

**Scheme 33:**
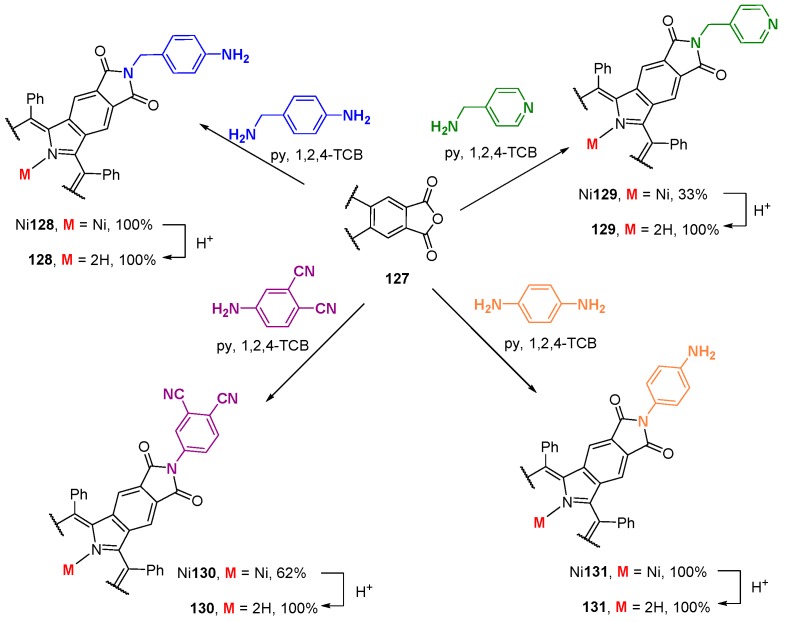
Reactions of anhydride **127** with alkylamines and arylamines affording “phthalimides” [158].

The reaction of anhydride **127** in the presence of adequate alkylamines and arylamines afforded the corresponding “phthalimides” **128**–**131** in moderate to excellent yields (Scheme 33) [158].

**Scheme 34:**
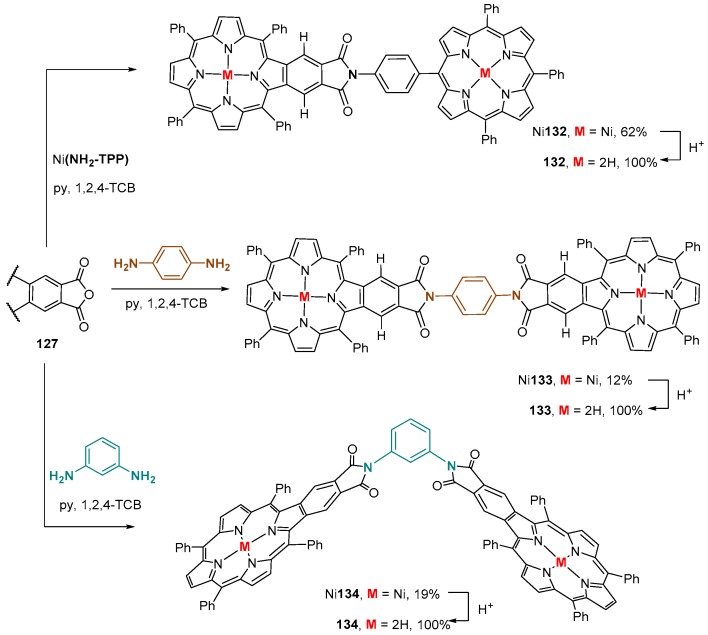
Synthetic access to bisphthalimides from anhydride **127** [158].

**Scheme 35:**
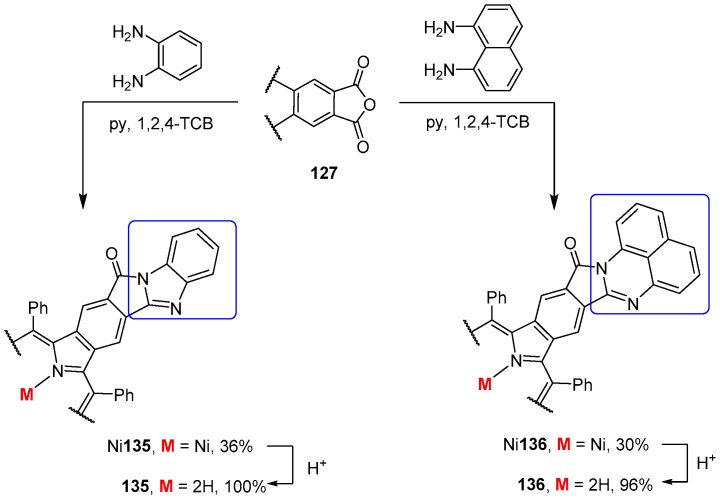
Reaction of anhydride **127** with benzene-1,2-diamine or naphthalene-1,8-diamine [158].

The reaction of **127** with a *meso*-(*p*-aminophenyl)porphyrin derivative, benzene-1,4-diamine or with benzene-1,3-diamine yielded the corresponding *N,N′*-(phenylene)bisphthalimides **132**, **133** and **134** (Scheme 34), whereas with benzene-1,2-diamine or naphthalene-1,8-diamine afforded the heterocyclic-fused porphyrins **135** and **136** (Scheme 35) [158]. Molecular mechanics simulations elucidate the multiplicity of signals observed in the NMR spectra of the *N*,*N′*-(1,4-phenylene)bisphthalimide **133**. This molecule exhibits two preferential conformations corresponding to a coplanar and an almost perpendicular arrangement of the benzoporphyrin units relative to the central benzene ring.

**Scheme 36:**
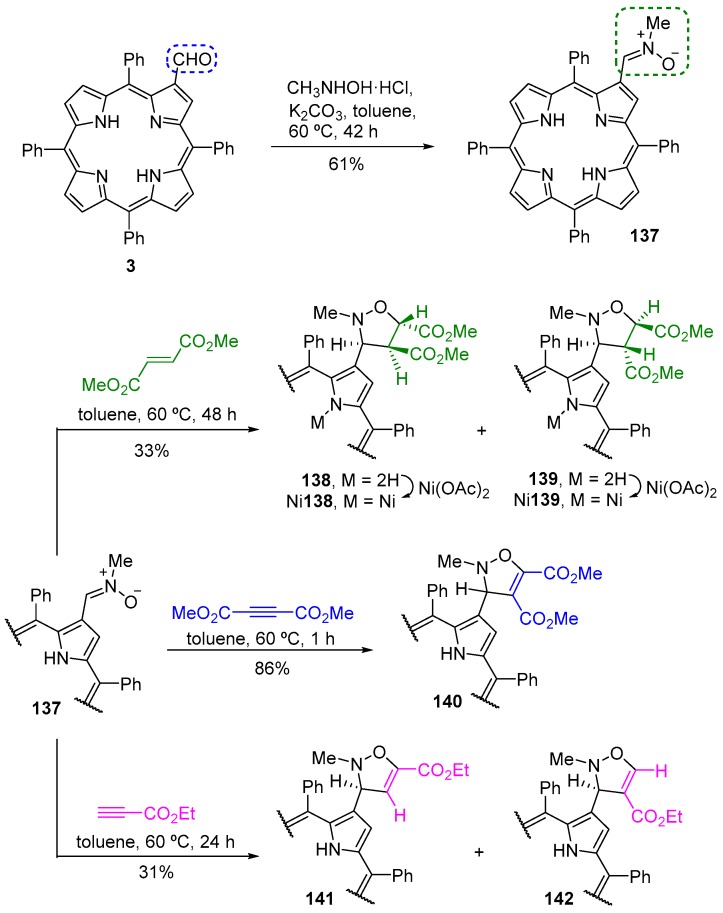
Synthesis of porphyrinyl nitrone **137** and its reaction with dipolarophiles [159].

As an extension of the previous studies on cycloaddition reactions with porphyrins, it was showed that the porphyrinyl nitrone **137**, isolated from the reaction of β-CHOTPP **3** with *N*-methyl-hydroxylamine hydrochloride, reacts with dimethyl fumarate, DMAD and ethyl propiolate to give the corresponding cycloadducts (Scheme 36) [159]. 

All reactions were performed in toluene at 60 °C and afforded the expected isoxazolidine and isoxazoline cycloadducts **138**–**142**. The best yield was obtained when dimethyl fumarate was used as the dipolarophile and the separation of the two diastereoisomeric adducts was possible after their metalation to Ni**138** and Ni**139**.

**Scheme 37:**
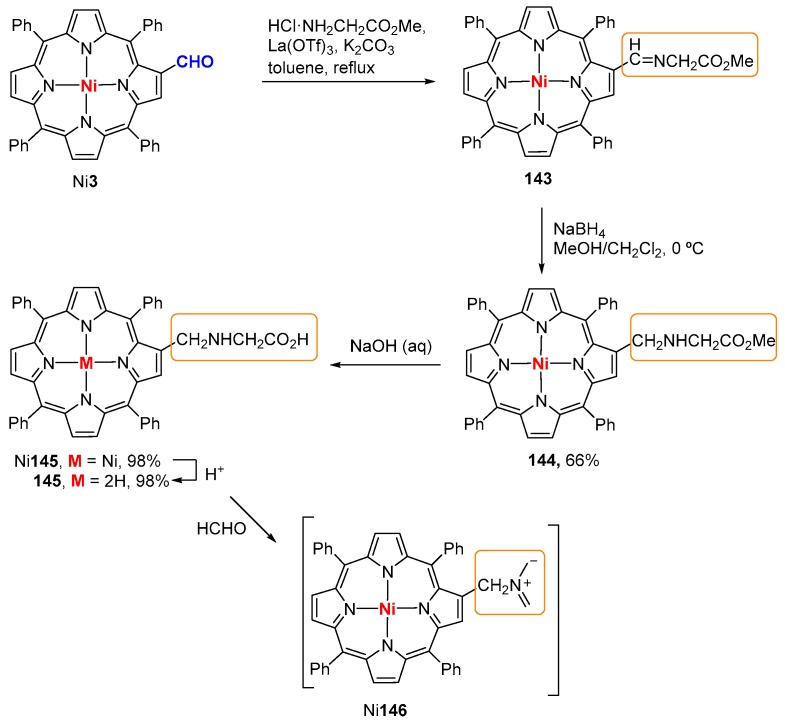
Synthesis of the azomethine ylide Ni**146** [160].

**Scheme 38:**
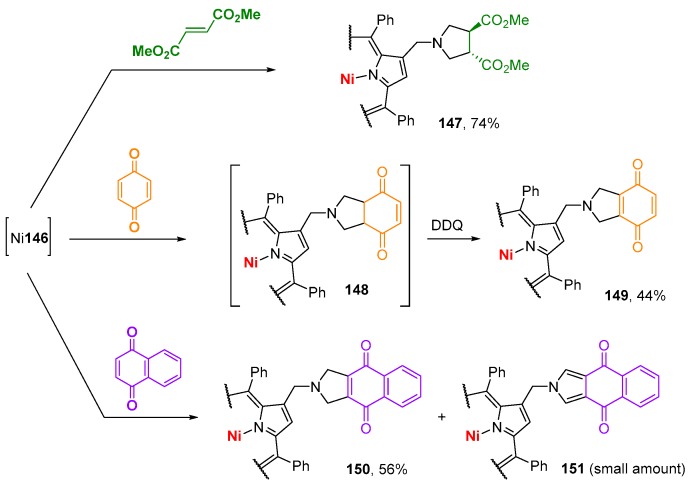
Reaction of the azomethine ylide Ni**146** with dipolarophiles [160].

**Scheme 39:**
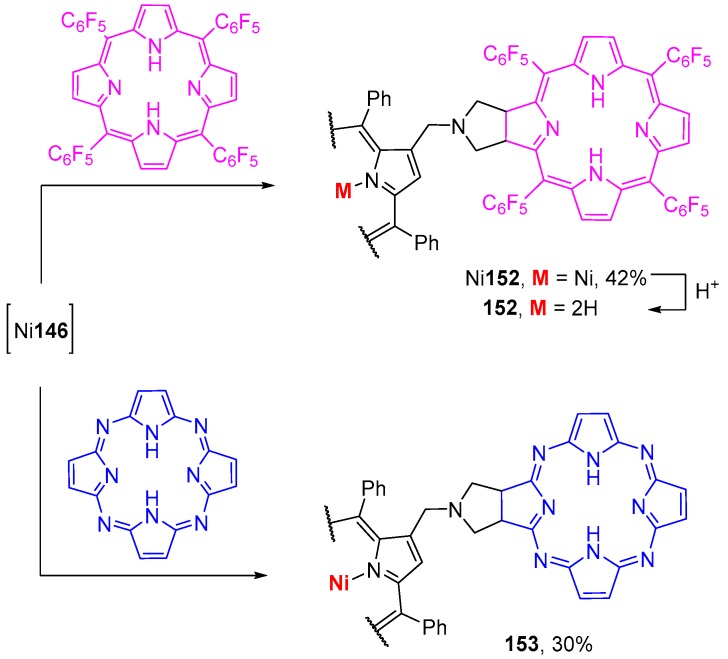
Reaction of the azomethine ylide **146a** with aromatic macrocycles [160].

The condensation of Ni**3** with glycine methyl ester hydrochloride, followed by reduction and hydrolysis afforded the *N-*(porphyrin-2-ylmethyl)glycine Ni**145** which after reaction with paraformaldehyde gave the azomethine ylide Ni**146** (Scheme 37) [160]. This 1,3-dipole reacted with dimethyl fumarate to give the expected adduct **147** while with 1,4-benzo-and 1,4-naphthoquinones only the dehydrogenated adducts **149** and **150**/**151** were isolated (Scheme 38). The reaction of the same ylide with *meso*-tetrakis(pentafluorophenyl)porphyrin and the tetraazaporphyrin gave access to the porphyrin–chlorins **152** and porphyrin–tetraazachlorin **153** dyads (Scheme 39).

**Figure 17:**
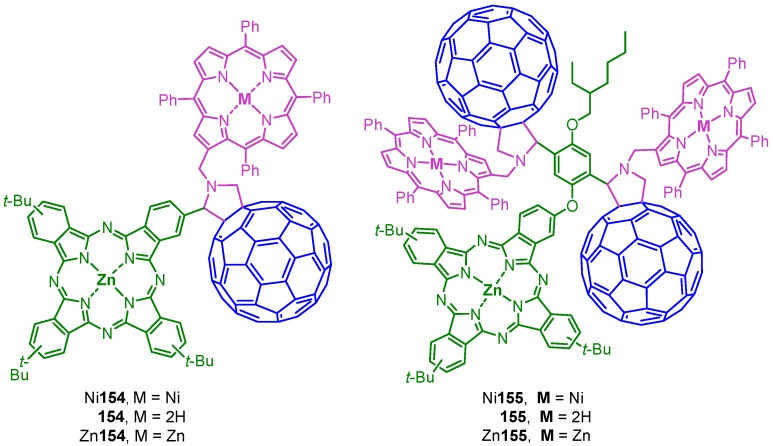
Fullerene-phthalocyanine-porphyrin triads and pentads [161].

The *N*-(porphyrin-2-ylmethyl)glycine Ni**145** was also used to generate phthalocyanine-substituted azomethine ylides that could be trapped with fullerene C_60_ to afford, in one step, the fullerene–phthalocyanine–porphyrin triads or pentads Ni**154** and Ni**155** (Figure 17) [161]. Demetallation and metalation of the porphyrin units with Zn(OAc)_2_ afforded the corresponding free-bases and Zn(II) complexes. These triads and pentads, upon excitation of their porphyrin components, give rise to a sequence of energy and charge transfer reactions yielding radical ion pair states.

## 7. Final Remarks

As shown in this review, porphyrin macrocycles containing simple substituents like the formyl one at pyrrolic positions provide versatile building blocks for the preparation of new significant derivatives through easy synthetic strategies. Examples of the latter are the reactions with methylene active compounds, amines and other nitrogen derivatives and with Wittig reagents. Further manipulation of some of the obtained derivatives, namely those bearing chalcone, imine/amine and vinyl units allows thee expansion to a plethora of new compounds. Other transformations like the 1,5-electrocyclic ring closure of porphyrinic azomethine ylides can give rise to pyrrolo[3,4-*b*]porphyrins and these can afford efficiently 1,3-dioxopyrrolo[3,4-*b*]porphyrins and anhydrides. The cycloaddition avenue can also lead to several derivatives with potential biological applications. The discovery of a simple access to Kröhnke type porphyrins and their potential as chemo-responsive materials deserves further exploration of their use, particularly in the construction of supramolecular systems. Finally the synthetic methodologies established with simple porphyrins can be extended to other tetrapyrrolic macrocycles; a wide range of new derivatives can be envisaged as well as their potential applications in solving many problems to the mankind.

## Figures and Tables

**Table 1:** Vilsmeier–Haack formylation of the Ni(II) and Cu(II) complexes of 5,10,15,20-tetraarylporphyrins under MW conditions.

Ar	M	MW Power (W)/Time (min)	Yield of Ni3 or Cu3 Under classical Heating Conditions (%)	Yield of Ni3 or Cu3 Under MW Heating Condition (%)
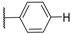	**Ni**	500/30	86	74
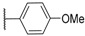			57	50
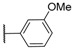			41	36
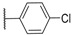			51	^a^
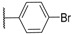			43	^a^
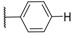	**Cu**	160/15	95	90
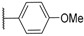			68	^a^
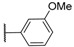			88	41
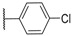			83	19
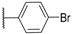			87	26

^a^ Not determined.

**Table 2:** Yields of the products obtained in the reaction of β-CHOTPP **3** with ketones **21**.

Entry	Ketone 21	Time (h)	22 (% Yield)	23 (% Yield)	24 (% Yield)
1 *^a^*	**a**	2	68	14	-
2	**b**	4	61	22	-
3	**c**	5.5	10	6	46
4 *^b^*	**c**	2.5	55	26	6
5	**d**	8	19	8	71
6 *^b^*	**d**	8	58	15	9
7	**e**	3	23	19	40
8 *^b^*	**e**	3	45	29	10

*^a^* Aryl methyl ketone (3 equiv.), La(OTf)_3_ (20 mol %), NH_4_OAc (4 equiv.), toluene, reflux; ^b^ Aryl methyl ketone (5 equiv.), La(OTf)_3_ (20 mol %), NH_4_OAc (8 equiv.), toluene, reflux.

## Abbreviations

1,2,4-TCB	1,2,4-trichlorobenzene
AcOH	acetic acid
AOT	sodium 1,4-bis(2-ethylhexyl)sulfosuccinate
DBSA	*p*-dodecylbenzenesulfonic acid
DDQ	2,3-dichloro-5,6-dicyano-1,4-benzoquinone
DFT	density functional theory
DMAD	dimethyl acetylenedicarboxylate
DMF	dimethylformamide
DPBF	1,3-diphenylisobenzofuran
ESI-MS	electrospray ionisation mass spectrometry
ESI-MS/MS	electrospray ionization tandem mass spectrometry
GC	gas chromatography
ISC	intersystem crossing
IUPAC	International Union of Pure and Applied Chemistry
MALDI-TOF-MS	matrix-assisted laser desorption/ionization time-of-flight mass spectrometry
min	minute
MS	mass spectrometry
MW	microwave
NiTPP	(5,10,15,20-tetraphenylporphyrinato)nickel(II)
NMR	nuclear magnetic resonance
*o*-DCB	1,2-dichlorobenzene
PDT	photodynamic therapy
PMMA	poly(methylmethacrylate)
TCNE	tetracyanoethylene
TFA	trifluoroacetic acid
THF	tetrahydrofuran
TPP	5,10,15,20-tetraphenylporphyrin
UV-vis	ultraviolet-visible
β-CHOTPP	2-formyl-5,10,15,20-tetrapenhylporphyrin
β-CHOTPP-*m*-MeOTPP	2-formyl-5,10,15,20-tetrakis(3-methoxyphenyl)porphyrin
β-vinylTPP	2-vinyl-5,10,15,20- tetraphenylporphyrin

## References

[1] García-Sánchez M.A., Rojas-González F., Menchaca-Campos E.C., Tello-Solís S.R., Quiroz-Segoviano R.I.Y., Diaz-Alejo L.A., Salas-Bañales E., Campero A. (2013). Crossed and linked histories of tetrapyrrolic macrocycles and their use for engineering pores within sol-gel matrices. Molecules.

[2] Fischer H., Zeile K. (1929). Synthese des hämatoporphyrins, protoporphyrins und hämins. Ann. Chem..

[3] Sheldon R.A. (1994). Metalloporphyrins in Catalytic Oxidations.

[4] Costas M. (2011). Selective C–H oxidation catalysed by metalloporphyrins. Coord. Chem. Rev..

[5] Costentin C., Robert M., Savéant J.-M. (2015). Current issues in molecular catalysis illustrated by iron porphyrins as catalysts of the CO_2_-to-CO electrochemical conversion. Acc. Chem. Res..

[6] Zucca P., Neves C.M.B., Simões M.M.Q., Neves M.G.P.M.S., Cocco G., Sanjust E. (2016). Immobilized lignin peroxidase-like metalloporphyrins as reusable catalysts in oxidative bleaching of industrial dyes. Molecules.

[7] Nakagaki S., Mantovani K.M., Machado G.S., Castro K.A.D.F., Wypych F. (2016). Recent advances in solid catalysts obtained by metalloporphyrins immobilization on layered anionic exchangers: A short review and some new catalytic results. Molecules.

[8] Rebelo S.L.H., Silva A.M.N., Medforth C.J., Freire C. (2016). Iron(III) fluorinated porphyrins: Greener chemistry from synthesis to oxidative catalysis reactions. Molecules.

[9] Jurow M., Schuckman A.E., Batteas J.D., Drain C.M. (2010). Porphyrins as molecular electronic components of functional devices. Coord. Chem. Rev..

[10] Wong K.-T., Bassani D.M. (2014). Energy transfer in supramolecular materials for new applications in photonics and electronics. NPG Asia Mater..

[11] Paolesse R., Nardis S., Monti D., Stefanelli M., Di Natale C. (2017). Porphyrinoids for chemical sensor applications. Chem. Rev..

[12] Auwärter W., Écija D., Klappenberger F., Barth J.V. (2015). Porphyrins at interfaces. Nat. Chem..

[13] Ding Y., Tang Y., Zhua W., Xie Y. (2015). Fluorescent and colorimetric ion probes based on conjugated oligopyrroles. Chem. Soc. Rev..

[14] Guan W., Zhou W., Lu J., Lu C. (2015). Luminescent films for chemo-and biosensing. Chem. Soc. Rev..

[15] Figueira F., Rodrigues J.M.M., Farinha A.A.S., Cavaleiro J.A.S., Tomé J.P.C. (2016). Synthesis and anion binding properties of porphyrins and related compounds. J. Porphyr. Phthalocyanines.

[16] Ding Y., Zhu W.-H., Xie Y. (2017). Development of ion chemosensors based on porphyrin analogues. Chem. Rev..

[17] Gust D., Moore T.A., Moore A.L. (2009). Solar fuels via artificial photosynthesis. Acc. Chem. Res..

[18] Li L.-L., Diau E.W.-G. (2013). Porphyrin-sensitized solar cells. Chem. Soc. Rev..

[19] Urbani M., Grätzel M., Nazeeruddin M.K., Torres T. (2014). Meso-substituted porphyrins for dye-sensitized solar cells. Chem. Rev..

[20] Pereira A.M.V.M., Cerqueira A.F.R., Moura N.M.M., Iglesias B.A., Faustino M.A.F., Neves M.G.P.M.S., Cavaleiro J.A.S., Lima M.J.C., da Cunha A.F. (2014). β-(*p*-Carboxyaminophenyl)porphyrin derivatives: New dyes for TiO_2_ dye-sensitized solar cells. J. Nanopart. Res..

[21] Birel1 Ö., Nadeem S., Duman H. (2017). Porphyrin-based Dye-Sensitized Solar Cells (DSSCs): A review. J. Fluoresc..

[22] Kundu S., Patra A. (2017). Nanoscale strategies for light harvesting. Chem. Rev..

[23] Ethirajan M., Chen Y., Joshi P., Pandey R.K. (2011). The role of porphyrin chemistry in tumor imaging and photodynamic therapy. Chem. Soc. Rev..

[24] Sheng H., Chaparro R.E., Sasaki T., Izutsu M., Pearlstein R.D., Tovmasyan A., Warner D.S. (2014). Metalloporphyrins as therapeutic catalytic oxidoreductants in central nervous system disorders. Antioxid. Redox Signal..

[25] Alves E., Faustino M.A.F., Neves M.G.P.M.S., Cunha Â., Nadais H., Almeida A. (2015). Potential applications of porphyrins in photodynamic inactivation beyond the medical scope. J. Photochem. Photobiol. C Photochem. Rev..

[26] Zhou Y.M., Liang X.L., Dai Z.F. (2016). Porphyrin-loaded nanoparticles for cancer theranostics. Nanoscale.

[27] Calvete M.J.F., Pinto S.M.A., Pereira M.M., Geraldes C.F.G.C. (2017). Metal coordinated pyrrole-based macrocycles as contrast agents for magnetic resonance imaging technologies: Synthesis and applications. Coord. Chem. Rev..

[28] Zhao J., Wu W., Sun J., Guo S. (2013). Triplet photosensitizers: From molecular design to applications. Chem. Soc. Rev..

[29] Xodo L.E., Cogoi S., Rapozzi V. (2016). Photosensitizers binding to nucleic acids as anticancer agents. Future Med. Chem..

[30] Abrahamse H., Hamblin M.R. (2016). New photosensitizers for photodynamic therapy. Biochem. J..

[31] Luo D., Carter K.A., Miranda D., Lovell J.F. (2017). Chemophototherapy: An emerging treatment option for solid tumors. Adv. Sci..

[32] Grin M.A., Mironov A.F. (2016). Chemical transformations of bacteriochlorophyll a and its medical applications. Russ. Chem. Bull..

[33] Grin M.A., Mironov A.F., Shtil A.A. (2008). Bacteriochlorophyll a and its derivatives: Chemistry and perspectives for cancer therapy. Anti-Cancer Agent Med. Chem..

[34] Marciel M., Teles L., Moreira B., Pacheco M., Lourenço L.M.O., Neves M.G.P.M.S., Tomé J.P.C., Faustino M.A.F., Almeida A. (2016). An effective and potentially safe blood disinfection protocol using tetrapyrrolic photosensitizers. Future Med. Chem..

[35] Luksiene Z. (2005). New approach to inactivation of harmful and pathogenic microorganisms by photosensitization. Food Technol. Biotechnol..

[36] Felgenträger A., Maisch T., Späth A., Schröder J.A., Bäumler W. (2014). Singlet oxygen generation in porphyrin-doped polymeric surface coating enables antimicrobial effects on Staphylococcus aureus. Phys. Chem. Chem. Phys..

[37] Jemli M., Alouini Z., Sabbahi S., Gueddari M. (2002). Destruction of fecal bacteria in wastewater by three photosensitizers. J. Environ. Monit..

[38] Carvalho C.M.B., Gomes A.T.P.C., Fernandes S.C.D., Prata A.C.B., Almeida M.A., Cunha M.A., Tomé J.P.C., Faustino M.A.F., Neves M.G.P.M.S., Tomé A.C. (2007). Photoinactivation of bacteria in wastewater by porphyrins: Bacterial β-galactosidase activity and leucine-uptake as methods to monitor the process. J. Photochem. Photobiol. B.

[39] Carvalho C.M.B., Alves E., Costa L., Tomé J.P.C., Faustino M.A.F., Neves M.G.P.M.S., Tomé A.C., Cavaleiro J.A.S., Almeida A., Cunha A. (2010). Functional cationic nanomagnet−porphyrin hybrids for the photoinactivation of microorganisms. ACS Nano.

[40] Arrojado C., Pereira C., Tomé J.P.C., Faustino M.A.F., Neves M.G.P.M.S., Tomé A.C., Cavaleiro J.A.S., Cunha A., Calado R., Gomes N.C.M. (2011). Applicability of photodynamic antimicrobial chemotherapy as an alternative to inactivate fish pathogenic bacteria in aquaculture systems. Photochem. Photobiol. Sci..

[41] Coppellottu O., Fabris C., Soncin M., Magaraggia M., Camerin M., Jori G., Guidolin L. (2012). Porphyrin photosensitised processes in the prevention and treatment of water-and vector-borne diseases. Curr. Med. Chem..

[42] Almeida J., Tomé J.P.C., Neves M.G.P.M.S., Tomé A.C., Cavaleiro J.A.S., Cunha A., Costa L., Faustino M.A.F., Almeida A. (2014). Photodynamic inactivation of multidrug-resistant bacteria in hospital wastewaters: Influence of residual antibiotics. Photochem. Photobiol. Sci..

[43] Behbahani A., Eghbali H., Ardjmand M., Noufal M.M.M., Williamson H.C., Sayar O. (2016). A novel bio-compatible sorbent based on carbon nanostructure modified by porphyrin for heavy metal separation from industrial wastewaters. J. Environ. Chem. Eng..

[44] Jaquinod L., Kadish K.M., Smith K.M., Guilard R. (2000). Functionalization of 5,10,15,20-tetra-substituted porphyrins. The Porphyrin Handbook.

[45] Vicente M.G.H., Kadish K.M., Smith K.M., Guilard R. (2000). Reactivity and functionalization of β-substituted porphyrins and chlorins. The Porphyrin Handbook.

[46] Tokuji S., Awane H., Yorimitsu H., Osuka A. (2013). Direct arylation of *meso*-formyl porphyrin. Chem. Eur. J..

[47] Vicente M.G.H., Smith K. (2014). Syntheses and functionalizations of porphyrin macrocycles. Curr. Org. Synth..

[48] Moss G.P. (1987). Nomenclature of tetrapyrroles (Recommendations 1986). Pure Appl. Chem..

[49] Rothemund P. (1935). Formation of porphyrins from pyrrole and aldehydes. J. Am. Chem. Soc..

[50] Rothemund P. (1936). A new porphyrin synthesis. The synthesis of porphin. J. Am. Chem. Soc..

[51] Rothemund P. (1939). Porphyrin studies. III. The structure of the porphine ring system. J. Am. Chem. Soc..

[52] Aronoff S., Calvin M. (1943). The porphyrin-like products of the reaction of pyrrole with benzaldehyde. J. Org. Chem..

[53] Calvin M., Ball R.H., Aronoff S. (1943). α,β,γ,δ-Tetraphenylchlorin. J. Am. Chem. Soc..

[54] Adler A.D., Longo F.R., Finarelli J.D., Goldmacher J., Assour J., Korsakoff L. (1967). A simplified synthesis for *meso*-tetraphenylporphin. J. Org. Chem..

[55] Barnett G.H., Hudson M.F., Smith K.M. (1975). Concerning *meso*-tetraphenylporphyrin purification. J. Chem. Soc. Perkin Trans. 1.

[56] Gonsalves A., Varejão J.M., Pereira M.M. (1991). Some new aspects related to the synthesis of *meso*-substituted porphyrins. J. Heterocycl. Chem..

[57] Gonsalves A.D.A.R., Pereira M.M. (1985). A new look into the Rothemund *meso*-tetraalkyl and tetraarylporphyrins synthesis. J. Heterocycl. Chem..

[58] Lindsey J.S., Hsu H.C., Schreiman I.C. (1986). Synthesis of tetraphenylporphyrins under very mild conditions. Tetrahedron Lett..

[59] Lindsey J.S., Schreiman I.C., Hsu H.C., Kearney P.C., Marguerettaz A.M. (1987). Rothemund and Adler–Longo reactions revisited: Synthesis of tetraphenylporphyrins under equilibrium conditions. J. Org. Chem..

[60] Lindsey J.S., Kadish K.M., Smith K.M., Guilard R. (2000). Synthesis of *meso*-substituted porphyrins. The Porphyrin Handbook.

[61] Sharghi H., Nejad A.H. (2003). Phosphorus pentachloride (PCl_5_) mediated synthesis of tetraarylporphyrins. Helv. Chim. Acta.

[62] Sharghi H., Nejad A.H. (2004). Novel synthesis of *meso*-tetraarylporphyrins using CF_3_SO_2_Cl under aerobic oxidation. Tetrahedron.

[63] Cavaleiro J.A.S., Tomé A.C., Neves M.G.P.M.S., Kadish K.M., Smith K.M., Guilard R. (2010). *Meso*-tetraarylporphyrin derivatives: New synthetic methodologies. Handbook of Porphyrin Science.

[64] Chauhan S., Sahoo B., Srinivas K. (2001). Microwave-assisted synthesis of 5,10,15,20-tetraaryl porphyrins. Synth. Commun..

[65] De Paula R., Faustino M.A., Pinto D.C., Neves M.G., Cavaleiro J.A. (2008). Kinetic study of *meso*-tetraphenylporphyrin synthesis under microwave irradiation. J. Heterocycl. Chem..

[66] Liu M.O., Tai C.-H., Wang W.-Y., Chen J.-R., Hu A.T., Wei T.-H. (2004). Microwave-assisted synthesis and reverse saturable absorption of phthalocyanines and porphyrins. J. Organomet. Chem..

[67] Nascimento B.F., Pineiro M., Rocha Gonsalves A.M.d.A., Ramos Silva M., Matos Beja A., Paixão J.A. (2007). Microwave-assisted synthesis of porphyrins and metalloporphyrins: A rapid and efficient synthetic method. J. Porphyr. Phthalocyanines.

[68] Henriques C.A., Pinto S., Aquino G.L., Pineiro M., Calvete M.J., Pereira M.M. (2014). Ecofriendly porphyrin synthesis by using water under microwave irradiation. Chem. Sustain. Chem..

[69] Neves M.G.P.M.S., Martins R.M., Tomé A.C., Silvestre A.J.D., Silva A.M.S., Félix V., Drew M.G.B., Cavaleiro J.A.S. (1999). *Meso*-substituted expanded porphyrins: New and stable hexaphyrins. Chem. Commun..

[70] Tanaka T., Osuka A. (2017). Chemistry of *meso*-aryl-substituted expanded porphyrins: Aromaticity and molecular twist. Chem. Rev..

[71] Costa L.D., Costa J.I.T., Tomé A.C. (2016). Porphyrin macrocycle modification: Pyrrole ring-contracted or-expanded porphyrinoids. Molecules.

[72] Inhoffen H.H., Fuhrhop J.-H., Voigt H., Brockmann H. (1966). Zur weiteren Kenntnis des Chlorophylls und des Hämins, VI. Formylierung der *meso*-kohlenstoffatome von alkyl-substituierten porphyrinen. Justus Liebigs Ann. Chem..

[73] Johnson A.W., Oldfield D. (1966). *Meso*-Substitution products of ætioporphyrin I. J. Chem. Soc. C.

[74] Inhoffen H.H., Buchler J.W., Thomas R. (1969). Zur weiteren kenntnis des chlorophylls und des hamins, XXV. 3, 4,7,8-Tetrahydro-octaäthylporphin (“Bacterio-octaäthylchlorin”). Tetrahedron. Lett..

[75] Callot H.J. (1973). Nouvelles voies d’accès aux vinylporphyrines. Tetrahedron.

[76] Callot H.J. (1973). Wittig reaction on some formylporphyrins-example of extremely facile deformylation. Bull. Soc. Chim. Fr..

[77] Callot H.J., Castro B., Selve C. (1978). Porphyrines synthetic ues porteuses de chaines laterales peptidiques. I Atropoisomérie d’amides cis(méso-tétraphénylporphyrinyl)-3-propénoïques. Tetrahedron. Lett..

[78] Momenteau M., Loock B., Bisagni E., Rougee M. (1979). Five-coordinate iron(II) porphyrins derived from *meso*-α,β,γ,δ-tetraphenylporphin: Synthesis, characterization, and coordinating properties. Can. J. Chem..

[79] Barloy L., Dolphin D., Dupré D., Wijesekera T.P. (1994). Anomalous double cyclization reactions of β-formylporphyrins. J. Org. Chem..

[80] Balakumar A., Mutukumaran K., Lindsey J.S. (2004). A New route to *meso*-formyl porphyrins. J. Org. Chem..

[81] Ponomarev G.V., Yashunskii D.V., Moskovkin A.S. (1997). Porphyrins. 35. Unusual course of the Vilsmeir reaction when formylating the platinum complex of octaethylporphyrin (revision of the known reaction). Chem. Heterocycl. Compd..

[82] Henrick K., Owston P.G., Peters R., Tasker P.A., Dell A. (1980). Cyclisation involving a *meso*-phenyl substituent of a metalloporphyrin: X-ray structure of 5,10,15-triphenyl{2^2^-oxo-benzo[2^3^2^4^]cyclohexa[*a,b*]porphinato(2-)} copper(II). Inorg. Chim. Acta.

[83] Callot H.J., Schaeffer E., Cromer R., Metz F. (1990). Unexpected routes to naphthoporphyrin derivatives. Tetrahedron.

[84] Bonfantini E.E., Burrell A.K., Campbell W.M., Crossley M.J., Gosper J.J., Harding M.M., Officer D.L., Reid D.C.W. (2002). Efficient synthesis of free-base 2-formyl-5,10,15,20-tetraarylporphyrins, their reduction and conversion to [(porphyrin-2-yl)methyl]phosphonium salts. J. Porphyrins. Phthalocyanines.

[85] Ponomarev G.V., Maravin G.B. (1982). Porphyrins. 14. Synthesis and properties of 1-substituted derivatives of 5,10,15,20-tetraphenylporphyrin. Chem. Heterocycl. Compd..

[86] Silva A.M.G., Faustino M.A.F., Silva T.M.P.C., Neves M.G.P.M.S., Tomé A.C., Silva A.M.S., Cavaleiro J.A.S. (2002). NMR characterisation of five isomeric β,β´-diformyl-*meso*-tetraphenylporphyrins. J. Chem. Soc. Perkin Trans. 1.

[87] Mironov A.F., Grin M.A., Moskalchuk T.V., Shashkov A.S., Lokshin B.V. (2002). Synthesis and unusual spectroscopic properties of diformyltetraarylporphyrins. Mendeleev Commun..

[88] Mironov A.F., Moskalchuk T.V., Shashkov A.S. (2004). Formylation reaction in series of *meso*-tetraaryl substituted porphyrins and chlorins. Russ. J. Bioorg. Chem..

[89] Yaseen M., Ali M., NajeebUllah M., Ali Munawar M., Khokhara I. (2009). Microwave-assisted synthesis, metallation, and Duff formylation of porphyrins. J. Heterocycl. Chem..

[90] Moura N.M.M., Faustino M.A.F., Neves M.G.P.M.S., Duarte A.C., Cavaleiro J.A.S. (2011). Vilsmeier-Haack formylation of Cu(II) and Ni(II) porphyrin complexes under microwaves irradiation. J. Porphyrins. Phthalocyanines.

[91] Rish I.G., Pshezhetskii V.S., Askarov K.A., Ponomarev G.V. (1985). Porphyrins. 20. Interaction of 2-formyl-5,10,15,20-tetraphenylporphyrin with CH acids. Chem. Heterocycl. Compd..

[92] Chen C.-T., Yeh H.-C., Zhang X., Yu J. (1999). Olefin-mediated interaction observed for nickel tetraphenylporphyrins with an acceptor substituted on the *β*-carbon. Org. Lett..

[93] Yeha H.-C., Chen C.-T., Yu J., Tsaic P.-C., Wang J.-K. (2007). Conformation and π-conjugation of olefin-bridged acceptor on the pyrrole β-carbon of nickel tetraphenylporphyrins: Implicit evidence from linear and nonlinear optical properties. J. Porphyrins. Phthalocyanines.

[94] Ramos C.I.V., Moura N.M.M., Santos S.M.F., Faustino M.A., Tomé J.P.C., Amado F.M.L., Neves M.G.P.M.S. (2015). An insight into the gas-phase fragmentations of potential molecular sensors with porphyrin-chalcone structures. Int. J. Mass Spectrom..

[95] Silva E.M.P., Domingues P., Tomé J.P.C., Faustino M.A.F., Neves M.G.P.M.S., Tomé A.C., Dauzonne D., Silva A.M.S., Cavaleiro J.A.S., Ferrer-Correia A.J. (2008). Electrospray tandem mass spectrometry of β-nitroalkenyl *meso*-tetraphenylporphyrins. Eur. J. Mass Spectrom..

[96] Giribabu L., Kumar C.V., Reddy P.Y. (2006). Porphyrin-rhodanine dyads for dye sensitized solar cells. J. Porphyrins. Phthalocyanines.

[97] Silva E.M.P., Giuntini F., Faustino M.A.F., Tomé J.P.C., Neves M.G.P.M.S., Tomé A.C., Silva A.M.S., Santana-Marques M.G., Ferrer-Correia A.J., Cavaleiro J.A.S. (2005). Synthesis of cationic *β*-vinyl substituted *meso*-tetraphenylporphyrins and their in vitro activity against herpes simplex virus type. Bioorg. Med. Chem. Lett..

[98] Burrel A.K., Officer D.L., Reid D.C.W., Wild K.Y. (1998). Controlling the structure of supramolecular porphyrin arrays. Angew. Chem. Int. Ed. Engl..

[99] Burrel A.K., Officer D.L. (1998). Functionalizing porphyrins via Wittig reactions: A building block approach. Synlett.

[100] Bonfantini E.E., Officer D.L. (1993). The synthesis of butadiene-bridged porphyrin dimers and styryl porphyrins using a porphyrin-derived Wittig reagent. Tetrahedron Lett..

[101] Izquierdo R.A., Barros C.M., Santana-Marques M.G., Ferrer-Correia A.J., Silva E.M.P., Giuntini F., Faustino M.A.F., Tomé J.P.C., Tomé A.C., Silva A.M.S. (2007). Characterization of isomeric cationic porphyrins with β-pyrrolic substituents by electrospray mass spectrometry: The singular behavior of a potential virus photoinactivator. J. Am. Soc. Mass Spectrom..

[102] Serra V.V., Andrade S.M., Silva E.M.P., Silva A.M.S., Neves M.G.P.M.S., Costa S.M.B. (2013). Structural effects of the β-vinyl linker in pyridinium porphyrins: Spectroscopic studies in organic solvents and AOT reverse micelles. J. Phys. Chem. B.

[103] Silva E.M.P., Ramos C.I.V., Pereira P.M.R., Giuntini F., Faustino M.A.F., Tomé J.P.C., Tomé A.C., Silva A.M.S., Santana-Marques M.G., Neves M.G.P.M.S. (2012). Cationic β-vinyl substituted *meso*-tetraphenylporphyrins: Synthesis and non-covalent interactions with a short poly(dGdC) duplex. J. Porphyrins. Phthalocyanines.

[104] Silva E.M.P., Serra V.V., Ribeiro A.O., Tomé J.P.C., Domingues P., Faustino M.A.F., Neves M.G.P.M.S., Tomé A.C., Cavaleiro J.A.S., Ferrer-Correia A.J. (2006). Characterization of cationic glycoporphyrins by electrospray tandem mass spectrometry. Rapid Commun. Mass Spectrom..

[105] Ishkov Y.V., Zhilina Z.I., Barday L.P. (2003). The interaction of formylporphyrins with weak CH-acids. J. Porphyrins. Phthalocyanines.

[106] Ishkov Y.V., Zhilina Z.I., Barday L.P., Vodzinskii S.V. (2004). Porphyrins and their derivatives: XXIII. Reaction of formylporphyrins with weak CH acids. Russ. J. Org. Chem..

[107] Moura N.M.M., Faustino M.A.F., Neves M.G.P.M.S., Paz F.A.A., Silva A.M.S., Tomé A.C., Cavaleiro J.A.S. (2012). A new synthetic approach to benzoporphyrins and Kröhnke type porphyrin-2-ylpyridines. Chem. Commun..

[108] Moura N.M.M., Ramos C.I.V., Linhares I., Santos S.M., Faustino M.A.F., Almeida A., Cavaleiro J.A.S., Amado F.M.L., Lodeiro C., Neves M.G.P.M.S. (2016). Synthesis, characterization and biological evaluation of cationic porphyrin-terpyridine derivatives. RSC Adv..

[109] Moura N.M.M., Nuñez C., Faustino M.A.F., Cavaleiro J.A.S., Neves M.G.P.M.S., Capelo J.L., Lodeiro C. (2014). Preparation and ion recognition features of porphyrin-chalcone type compounds as efficient red-fluorescent materials. J. Mater. Chem. C.

[110] Fleischer E.B., Dixon F. (1977). Definitive evidence for the existence of the “sitting-atop” porphyrin complexes in nonaqueous solutions. Bioinorg. Chem..

[111] Callot H.J., Chevrier B., Weiss R. (1979). Sitting-atop porphyrin complexes. The structure of the bischloromercury(II) complex of *N*-tosylaminooctaethylporphyrin. J. Am. Chem. Soc..

[112] Moura N.M.M., Nuñez C., Santos S.M., Faustino M.A.F., Cavaleiro J.A.S., Neves M.G.P.M.S., Capelo J.L., Lodeiro C. (2014). Synthesis, spectroscopy studies, and theoretical calculations of new fluorescent probes based on pyrazole containing porphyrins for Zn(II), Cd(II), and Hg(II) optical detection. Inorg. Chem..

[113] Arnold D.P., Nitschinsk L.J. (1992). Porphyrin dimers linked by conjugated butadiynes. Tetrahedron.

[114] Gosper J.J., Ali M. (1994). A conformationally constrained conjugated porphyrin dimer. J. Chem. Soc. Chem. Commun..

[115] Arnold D.P., Gaete-Holmes R., Johnson A.W., Smith A.R.P., Williams G.A. (1978). Wittig condensation products from nickel *meso*-formyl-octaethyl-porphyrin and -aetioporphyrin I and some cyclisation reactions. J. Chem. Soc. Perkin Trans. 1.

[116] Burrell A.K., Officer D.L., Plieger P.G., Reid D.C.W. (2001). Synthetic routes to multiporphyrin arrays. Chem. Rev..

[117] Faustino M.A., Neves M.G.P.M.S., Vicente M.G.H., Silva A.M.S., Cavaleiro J.A.S. (1995). New naphthochlorins from the intramolecular cyclization of β-*vinyl-meso*-tetraarylporphyrins. Tetrahedron Lett..

[118] Silva A.M.G., de Oliveira K.T., Faustino M.A.F., Neves M.G.P.M.S., Tomé A.C., Silva A.M.S., Cavaleiro J.A.S., Brandão P., Felix V. (2008). Chemical transformations of mono- and bis(buta-1,3-dien-1-yl)porphyrins: A new synthetic approach to mono- and dibenzoporphyrins. Eur. J. Org. Chem..

[119] Grigg R., Johnson A.W., Sweeney A. (1968). Diels–Alder additions to protoporphyrin IX dimethyl ester. J. Chem. Soc. Chem. Commun..

[120] Callot H.J., Johnson A.W., Sweeney A. (1973). Additions to porphins involving the formation of new carbon-carbon bonds. J. Chem. Soc. Perkin Trans. 1.

[121] DiNello R.K., Dolphin D. (1980). Reactions of protoporphyrin with tetracyanoethylene. J. Org. Chem..

[122] Morgan A.R., Pangka V.S., Dolphin D. (1984). Ready syntheses of benzoporphyrins via Diels–Alder reactions with protoporphyrin IX. J. Chem. Soc. Chem. Commun..

[123] Pangka V.S., Morgan A.R., Dolphin A. (1986). The Diels–Alder reactions of protoporphyrin IX dimethyl ester with electron-deficient alkynes. J. Org. Chem..

[124] Yon-Hin P., Wijesekera T.P., Dolphin D. (1989). Transformation of a monovinylporphyrin to benzoporphyrins via Diels–Alder adducts. Tetrahedron Lett..

[125] Inhoffen H.H., Brockmann H., Bliesener K.-M. (1969). Zur weiteren Kenntnis des Chlorophylls und des Hamins, XXX. Photoprotoporphyrine und ihre Umwandlung in Spirographis-sowie Isospirographis-porphyrin. Liebigs. Ann. Chem..

[126] De Oliveira K.T., Silva A.M.S., Tomé A.C., Neves M.G.P.M.S., Neri C.R., Garcia V.S., Serra O.A., Iamamoto Y., Cavaleiro J.A.S. (2008). Synthesis of new amphiphilic chlorin derivatives from protoporphyrin-IX dimethyl ester. Tetrahedron.

[127] Petrilli R., Praça F.S.G., Carollo A.R.H., Medina W.S.G., de Oliveira K.T., Fantini M.C.A., Neves M.G.P.M.S., Cavaleiro J.A.S., Serra O.A., Iamamoto Y. (2013). Nanoparticles of lyotropic liquid crystals: A novel strategy for the topical delivery of a chlorin derivative for photodynamic therapy of skin cancer. Curr. Nanosci..

[128] Morgan A.R., Kohli D.H. (1995). Diels-Alder reactions of protoporphyrin ix dimethyl ester. Tetrahedron Lett..

[129] Cavaleiro J.A.S., Neves M.G.P.M.S., Tomé A.C. (2003). Cycloaddition reactions of porphyrins. ARKIVOC.

[130] Cavaleiro J.A.S., Jackson A.H., Neves M.G.P.M.S., Rao K.R.N. (1985). Diels–Alder reactions of protoporphyrin dimethyl esters with nitrosobenzenes; a novel degradation to formyl porphyrins. J. Chem. Soc. Chem. Commun..

[131] Faustino M.A.F., Neves M.G.P.M.S., Vicente M.G.H., Silva A.M.S., Cavaleiro J.A.S. (1996). Diels–Alder reactions of Ni(II) β-vinyl-*meso*-tetraarylporphyrins. Tetrahedron Lett..

[132] Faustino M.A.F., Neves M.G.P.M.S., Silva A.M.S., Cavaleiro J.A.S. (1997). A new *meso*-tetraphenylchlorin and meso-tetraphenylbenzoporphyrin from Diels–Alder reactions of Ni(II) β-vinyl *meso*-tetraphenylporphyrin. Chimia.

[133] Matsumoto K., Kimura S. (2000). Diels–Alder reaction of Ni(II) β-vinyl-*meso*-tetraphenylporphyrin with dimethyl acetylenedicarboxylate and *N*-phenyl maleimide. Heterocycl. Commun..

[134] Faustino M.A.F., Neves M.G.P.M.S., Tomé A.C., Silva A.M.S., Cavaleiro J.A.S. (2005). Diels–Alder reactions of beta-vinyl-*meso*-tetraphenylporphyrin with quinones. ARKIVOC.

[135] Matsumoto K., Kimura S., Morishita T., Misumi Y., Hayashi N. (2000). Diels–Alder reaction of Ni(II) β-vinyl-*meso*-tetraphenylporphyrin: A general method for synthesis of functionalized porphyrins. Synlett.

[136] Menezes J.C.J.M.D.S., Gomes A.T.P.C., Silva A.M.S., Faustino M.A.F., Neves M.G.P.M.S., Tomé A.C., da Silva F.C., Ferreira V.F., Cavaleiro J.A.S. (2011). Reaction of β-vinyl-*meso*-tetraphenylporphyrin with *o*-quinone methines. Synlett.

[137] Santos C.I.M., Oliveira E., Menezes J.C.J.M.D.S., Barata J.F.B., Faustino M.A.F., Ferreira V.F., Cavaleiro J.A.S., Neves M.G.P.M.S., Lodeiro C. (2014). New coumarin-corrole and -porphyrin conjugate multifunctional probes for anionic or cationic interactions: Synthesis, spectroscopy, and solid supported studies. Tetrahedron.

[138] Santos C.I.M., Oliveira E., Santos H.M., Menezes J.C.J.M.D.S., Faustino M.A.F., Cavaleiro J.A.S., Capelo J.L., Neves M.G.P.M.S., Lodeiro C. (2015). Untangling interactions of a zinc(II) complex containing a coumarin-porphyrin unit with alkaloids in water solutions: A photophysical study. Photochem. Photobiol. Sci..

[139] Menezes J.C.J.M.D.S., Faustino M.A.F., de Oliveira K.T., Uliana M.P., Ferreira V.F., Hackbarth S., Röder B., Tasso T.T., Furuyama T., Kobayashi N. (2014). Synthesis of new chlorin e6 trimethyl and protoporphyrin IX dimethyl ester derivatives and their photophysical and electrochemical characterizations. Chem. Eur. J..

[140] Cardoso M.F.C., Gomes A.T.P.C., Silva V.L.M., Silva A.M.S., Neves M.G.P.M.S., da Silva F.C., Ferreira V.F., Cavaleiro J.A.S. (2015). Ohmic heating assisted synthesis of coumarinyl porphyrin derivatives. RSC Adv..

[141] Cerqueira A.F.R., Almodôvar V.A.S., Neves M.G.P.M.S., Tomé A.C. (2017). Coumarin-tetrapyrrolic macrocycle conjugates: Synthesis and applications. Molecules.

[142] Silva Vera L.M., Santos Luis M.N.B.F., Silva Artur M.S. (2017). Ohmic heating: An emerging concept in organic synthesis. Chem. Eur. J..

[143] Moura N.M.M., Faustino M.A.F., Neves M.G.P.M.S., Tomé A.C., Rakib E.M., Hannioui A., Mojahidi S., Hackbarth S., Röder B., Paz F.A.A. (2012). Novel pyrazoline and pyrazole porphyrin derivatives: Synthesis and photophysical properties. Tetrahedron.

[144] Sharma S., Nath M. (2012). An efficient synthetic approach to novel nickel(II) 2-benzazolo-5,10,15,20-tetraphenylporphyrins. J. Heterocycl. Chem..

[145] Tekuri C., Singh D.K., Nath M. (2016). Synthesis, characterization and optical properties of β-substituted pyrrolo- and indolo[1,2-*a*]quinoxalinoporphyrins. Dyes Pigment..

[146] Giribabu L., Reeta P.S., Kanaparthi R.K., Srikanth M., Soujanya Y. (2013). Bis(porphyrin)-anthraquinone triads: Synthesis, spectroscopy, and photochemistry. J. Phys. Chem. A.

[147] Bastos M.M., Gomes A.T.P.C., Neves M.G.P.M.S., Silva A.M.S., Santos-Filho O.A., Boechat N., Cavaleiro J.A.S. (2013). Synthesis of β-substituted porphyrin derivatives containing heterocyclic moieties as potential photosensitizers against cutaneous Leishmaniasis. Eur. J. Org. Chem..

[148] Alonso C.M.A., Neves M.G.P.M.S., Tomé A.C., Silva A.M.S., Cavaleiro J.A.S. (2004). β-Imino-*meso*-tetraphenylporphyrin derivatives in hetero-Diels–Alder reactions. Eur. J. Org. Chem..

[149] Alonso C.M.A., Neves M.G.P.M.S., Tomé A.C., Silva A.M.S., Cavaleiro J.A.S. (2006). Beta-imino-*meso*-tetraphenylporphyrin derivatives: Reactivity towards ethyl diazoacetate. Jordan J. Chem..

[150] Drovetskaya T., Reed C.A., Boyd P. (1995). A fullerene-porphyrin conjugate. Tetrahedron Lett..

[151] Silva A.M.G., Tomé A.C., Neves M.G.P.M.S., Silva A.M.S., Cavaleiro J.A.S. (2002). Synthesis of new β-substituted *meso*-tetraphenylporphyrins via 1,3-dipolar cycloaddition reactions. 1. J. Org. Chem..

[152] Silva A.M.G., Faustino M.A.F., Tomé A.C., Neves M.G.P.M.S., Silva A.M.S., Cavaleiro J.A.S. (2001). A novel approach to the synthesis of mono- and dipyrroloporphyrins. J. Chem. Soc. Perkin Trans. 1.

[153] Jaquinod L., Gros C., Olmstead M.M., Antolovich M., Smith K.M. (1996). First syntheses of fused pyrroloporphyrins. Chem. Commun..

[154] Knapp S., Vasudevan J., Emge T.J., Arison B.H., Potenza J.A., Schugar H.J. (1998). A tethered porphyrin dimer with π overlap of a single pyrrole ring. Angew. Chem. Int. Ed..

[155] Vicente M.G.H., Jaquinod L., Khoury R.G., Madrona A.Y., Smith K.M. (1999). Synthesis and chemistry of new benzoporphyrins. Tetrahedron Lett..

[156] Liu W., Fronczek F.R., Vicente M.G.H., Smith K.M. (2005). Diels–Alder reactions of pyrrolo[3,4-*b*]porphyrins. Tetrahedron Lett..

[157] Carvalho C.M.B., Neves M.G.P.M.S., Tomé A.C., Paz F.A.A., Silva A.M.S., Cavaleiro J.A.S. (2011). 1,3-dioxopyrrolo[3,4-*b*]porphyrins: Synthesis and chemistry. Org. Lett..

[158] Carvalho C.M.B., Santos S.M., Neves M.G.P.M.S., Tomé A.C., Silva A.M.S., Rocha J., Cavaleiro J.A.S. (2013). *Meso*-tetraphenylbenzoporphyrin-2(2),2(3)-dicarboxylic anhydride: A platform to benzoporphyrin derivatives. J. Org. Chem..

[159] Silva A.F.F., Barata J.F.B., Silva A.M.G., Neves M.G.P.M.S., Tomé A.C., Silva A.M.S., Cavaleiro J.A.S. (2015). Reactivity of tetrapyrrolyl nitrones towards dipolarophiles bearing electron-withdrawing groups. Tetrahedron Lett..

[160] Silva A.M.G., Lacerda P.S.S., Tomé A.C., Neves M.G.P.M.S., Silva A.M.S., Cavaleiro J.A.S., Makarova E.A., Lukyanets E.A. (2006). Porphyrins in 1,3-dipolar cycloaddition reactions. Synthesis of new porphyrin-chlorin and porphyrin-tetraazachlorin dyads. J. Org. Chem..

[161] Enes R.F., Cid J.-J., Hausmann A., Trukhina O., Gouloumis A., Vázquez P., Cavaleiro J.A.S., Tomé A.C., Guldi D.M., Torres T. (2012). Synthesis and photophysical properties of fullerene-phthalocyanine-porphyrin triads and pentads. Chem. Eur. J..

